# Transcriptome-Wide Characterization of Alkaloids and Chlorophyll Biosynthesis in Lotus Plumule

**DOI:** 10.3389/fpls.2022.885503

**Published:** 2022-05-23

**Authors:** Heng Sun, Heyun Song, Xianbao Deng, Juan Liu, Dong Yang, Minghua Zhang, Yuxin Wang, Jia Xin, Lin Chen, Yanling Liu, Mei Yang

**Affiliations:** ^1^Aquatic Plant Research Center, Wuhan Botanical Garden, Chinese Academy of Sciences, Wuhan, China; ^2^Hubei Key Laboratory of Wetland Evolution and Ecological Restoration, Wuhan Botanical Garden, Chinese Academy of Sciences, Wuhan, China; ^3^University of Chinese Academy of Sciences, Beijing, China; ^4^Center of Applied Biotechnology, Wuhan Institute of Bioengineering, Wuhan, China

**Keywords:** lotus plumule, bisbenzylisoquinoline alkaloids, chlorophyll, biosynthetic mechanism, transcriptome analysis

## Abstract

Lotus plumule is a green tissue in the middle of seeds that predominantly accumulates bisbenzylisoquinoline alkaloids (bis-BIAs) and chlorophyll (Chl). However, the biosynthetic mechanisms of these two metabolites remain largely unknown in lotus. This study used physiological and RNA sequencing (RNA-Seq) approaches to characterize the development and molecular mechanisms of bis-BIAs and Chl biosynthesis in lotus plumule. Physiological analysis revealed that exponential plumule growth occurred between 9 and 15 days after pollination (DAP), which coincided with the onset of bis-BIAs biosynthesis and its subsequent rapid accumulation. Transcriptome analysis of lotus plumule identified a total of 8,725 differentially expressed genes (DEGs), representing ~27.7% of all transcripts in the lotus genome. Sixteen structural DEGs, potentially associated with bis-BIAs biosynthesis, were identified. Of these, 12 encoded *O*-methyltransferases (OMTs) are likely involved in the methylation and bis-BIAs diversity in lotus. In addition, functionally divergent paralogous and redundant homologous gene members of the BIAs biosynthesis pathway, as well as transcription factors co-expressed with bis-BIAs and Chl biosynthesis genes, were identified. Twenty-two genes encoding 16 conserved enzymes of the Chl biosynthesis pathway were identified, with the majority being significantly upregulated by Chl biosynthesis. Photosynthesis and Chl biosynthesis pathways were simultaneously activated during lotus plumule development. Moreover, our results showed that light-driven Pchlide reduction is essential for Chl biosynthesis in the lotus plumule. These results will be useful for enhancing our understanding of alkaloids and Chl biosynthesis in plants.

## Introduction

Lotus is a perennial aquatic plant in the family Nelumbonaceae that contains a single genus, *Nelumbo*, with two extant species: *Nelumbo nucifera* Gaertn. and *Nelumbo lutea* Pers (Wang et al., 2013). In Asia, lotus is an old domesticated herbaceous crop with versatile uses that are classified as seed-, rhizome-, and flower-lotus based on varieties (Yang et al., 2015). Lotus seed is not only an important reproductive organ consisting of the pericarp, seed coat, cotyledon, and plumule, but is also a rich source of nutrients and bioactive compounds with medicinal properties. The plumule, also known as *Lianzixin*, is a common traditional Chinese medicine with important pharmacological properties, such as antihypertensive, antiarrhythmic, and diuretic (Liu et al., 2017).

Alkaloids are a class of alkaline organic nitrogen compounds in plants (Liu et al., 2019). Lotus tissues, such as leaf, plumule, and petal are rich in benzylisoquinoline alkaloids (BIAs) (Deng et al., 2016). Bisbenzylisoquinoline alkaloids (bis-BIAs) are structural dimers of 1-benzylisoquinolines and are important bioactive components that are predominantly accumulated in the lotus plumule especially liensinine, isoliensinine, and neferineare (Deng et al., 2016). Previous studies have only focused on the identification, separation, purification, and pharmacological effects of bis-BIAs in lotus plumule (Deng et al., 2016; Chen et al., 2019; Liu et al., 2019). However, the molecular mechanisms underlying the biosynthesis of bis-BIAs in the lotus plumule remain largely unknown. BIAs are synthesized through a common pathway derived from the L-tyrosine substrate in plants. The substrate is subsequently catalyzed by tyrosine/DOPA decarboxylase (TYDC), norcoclaurine synthases (NCS), norcoclaurine 6-O-methyltransferase (6OMT), coclaurine N-methyltransferase (CNMT), (*S*)-N-methylcoclaurine-3′-hydroxylase (CYP80B), and 3′-hydroxy-N-methylcoclaurine 4′-O-methyltransferase (4′OMT) to produce (*S*)-reticuline, which is the common precursor of most BIAs (Ziegler and Facchini, 2008; Hagel and Facchini, 2013). In addition, bis-BIAs are produced *via* the catalysis of *N*-methylcoclaurine by the P450 enzyme CYP80A1 (Ziegler and Facchini, 2008). Notwithstanding, the alkaloids biosynthetic pathways vary greatly in different plants; thus, identification of key structural genes and determination of the biosynthetic mechanism of bis-BIAs in lotus plumule is necessary.

Unlike in many angiosperms, lotus plumule displays a dim-light photosynthetic capacity and can synthesize Chl while still being enclosed by dense layers of seed integuments, such as pericarp, seed coat, and cotyledon (Shen-Miller, 2007). As the most abundant pigment in the plant kingdom, Chl is crucial for light harvesting and energy transduction during photosynthesis (Tripathy and Pattanayak, 2012). The Chl biosynthetic pathway has been well-elucidated in higher plants, with over 16 enzymes and enzymatic steps responsible for this process identified and characterized (Tripathy and Pattanayak, 2012). Chl is synthesized through a complex pathway derived from the biosynthesis of the 5-aminolevulinic acid (ALA) precursor. Of the 16 enzymes reported to be involved in Chl biosynthesis, the conversion of protochlorophyllide (Pchlide) to chlorophyllide (Chlide) by the light-dependent Pchlide oxidoreductase (LPOR) is the only light-requiring reaction in angiosperms (Yamamoto et al., 2017). The LPOR encoding genes are nuclear-encoded and are distributed throughout oxygenic photosynthetic organisms. In contrast, gymnosperms employ an alternative Pchlide reduction reaction catalyzed by light-independent Pchlide oxidoreductase (DPOR) (Reinbothe et al., 2010). DPOR is encoded in the chloroplast genome by three genes, *chlL, chlN*, and *chlB*. To date, little is still known about Chl biosynthesis in basal eudicots including lotus. The plumule provides a model system for studying and improving our understanding of the mechanism of Chl biosynthesis in lotus and other basal eudicots.

The RNA-Seq has become a popular tool for uncovering the underlying molecular mechanisms of biological processes, including development, stress response, and metabolism processes in recent years (Fracasso et al., 2016; Goyal et al., 2016; Yang et al., 2017; Lanver et al., 2018; Xia et al., 2020; Sun et al., 2021). For example, the molecular mechanism of alkaloids biosynthesis has been clarified in numerous plants using RNA-seq (Guo et al., 2013; Cui et al., 2015; He et al., 2017; Deng et al., 2018). Due to its efficiency, this study used RNA-Seq technology to reveal the dynamic changes in gene expression during lotus plumule development. The result showed significant variations in alkaloid contents during plumule development and identification of structural genes likely associated with bis-BIAs biosynthesis, which were analyzed. In addition, the results clarified that the light-dependent Chl biosynthetic pathway in the lotus plumule. This study will expand our understanding of BIAs and Chl biosynthesis in plants.

## Materials and Methods

### Lotus Plumule Collection

Lotus cultivars were grown in the experimental field at Wuhan Botanical Garden (Wuhan, China). Lotus plumules were collected at 9, 12, 15, and 18 DAP from the cultivar “Jianxuan17” (JNP) and at 12, 15, and 18 DAP from the cultivar “China Antique” (CNP). Samples were immediately frozen in liquid nitrogen and then stored at −80°C until use.

### RNA Extraction and Sequencing

The plumule samples were ground into powder in liquid nitrogen, and total RNA was extracted using the Plant Total RNA Isolation Kit (Beijing Zoman Biotechnology Co., Ltd., Beijing, China). Illumina platform was used to sequence 21 high-quality RNA libraries at Biomarker Technologies Corporation (Beijing, China). The resulting clean data has been deposited at the National Center for Biotechnology Information (NCBI) with accession number, PRJNA747903.

### Analysis of RNA Sequencing Data

After removing adaptors and low-quality sequence reads, clean reads were mapped to the lotus reference genome sequence (Ming et al., 2013). The fragments per kilobase of transcript per million fragments mapped (FPKM) was calculated to quantify gene expression levels, and genes that met the Fold Change (FC) ≥ 2 and False Discovery Rate (FDR) <0.01 criteria were assigned as differentially expressed (DEGs).

Kyoto Encyclopedia of Genes and Genomes (KEGG) enrichment analysis was performed by KOBAS 3.0, and Gene Ontology (GO) enrichment analysis was implemented by the GOseq R packages (Bu et al., 2021). K-means analysis of gene expression was performed using Genesis software (Sturn et al., 2002). Principal component analysis (PCA), correlation analysis of libraries, and gene expression between the 510 TFs and 8,725 DEGs identified were performed using BMKCloud programs at www.biocloud.net. TFs were identified using PlantTFDB v5.0 (http://planttfdb.gao-lab.org/). Venn diagram, gene chromosomal location, synteny analysis, and heatmaps were visualized with TBtools software (Chen et al., 2020). The phylogenetic tree was constructed using MEGA7 software (Kumar et al., 2016). All protein sequences of bis-BIAs and Chl biosynthesis genes are listed in Supplementary Table 1.

### qRT-PCR Analysis

High-quality RNAs were reverse transcribed to cDNA using TransScript One-Step gDNA Removal and cDNA Synthesis SuperMix Kit (Lot#M31212, Beijing TransGen Biotech Co., Ltd., Beijing, China). Primers were designed using Primer Premier 5.0 and synthesized commercially (Huayu Gene, Wuhan, China). The qRT-PCR was performed using StepOnePlus Real-time PCR System (Applied Biosystems, USA) according to the protocol described by Deng et al. (2018). The *NnACTIN* (Gene ID NNU_24864) was used as the internal control to normalize the gene expression level. All primer sequences used are listed in Supplementary Table 2.

### Measurement of Alkaloid Content

Extraction and quantification of alkaloids in the lotus plumule were performed according to the protocol described by Deng et al. (2016). Briefly, fresh lotus plumule samples were ground to a fine powder in liquid nitrogen followed by extraction of alkaloids using 0.3 M HCl-methanol, 1:1, v/v extraction buffer. Quantification of alkaloid extracts was performed using high-performance liquid chromatography (HPLC, Agilent Technologies, USA).

### Measurement of Chl Content

Chl extraction and quantification were performed as previously described (Morley et al., 2020). Fresh lotus plumule samples were ground to a fine powder using liquid nitrogen followed by Chl extraction using 80% aqueous acetone. The absorption wavelength was set to 663 nm (A663) and 646 nm (A646), and detection was performed with an Infinite M200 Luminometer (Tecan, Mannerdorf, Switzerland). Chlorophyll *a* and Chlorophyll *b* were calculated according to the following equations, which were then summed to represent the total leaf Chl content.


$$\begin{array}{l} \text{Chlorophyll }\text{a}=12.21{\;}^{*}\;A663-2.81{\;}^{*}\;A646\\ \text{Chlorophyll }\text{b}=20.13{\;}^{*}\;A646-5.03{\;}^{*}\;A663 \end{array}$$

### Light and Dark Treatment

The seed-lotus cultivar, “Jianxuan17,” was grown in the experimental field at Wuhan Botanical Garden (Wuhan, China). Aluminum foil was then used to tightly wrap pods at 3 DAPs in July, and then, the procedure was repeated in August. The unwrapped pods were used as a control group. Pods were collected at 12, 15, and 18 DAP to analyze the effects of light/dark treatment on Chl biosynthesis in the lotus plumule.

### Statistical Analysis

Physiological data were statistically assessed by one-way ANOVA *via* IBM SPSS Statistics 20.0 software (SPSS Inc, USA), and significant differences in means were assessed with the least significant difference (LSD) test at *p* = 0.05.

## Results

### Morphological Changes, Chlorophyll, and Alkaloids Content During Lotus Plumule Development

**Figure 1:**
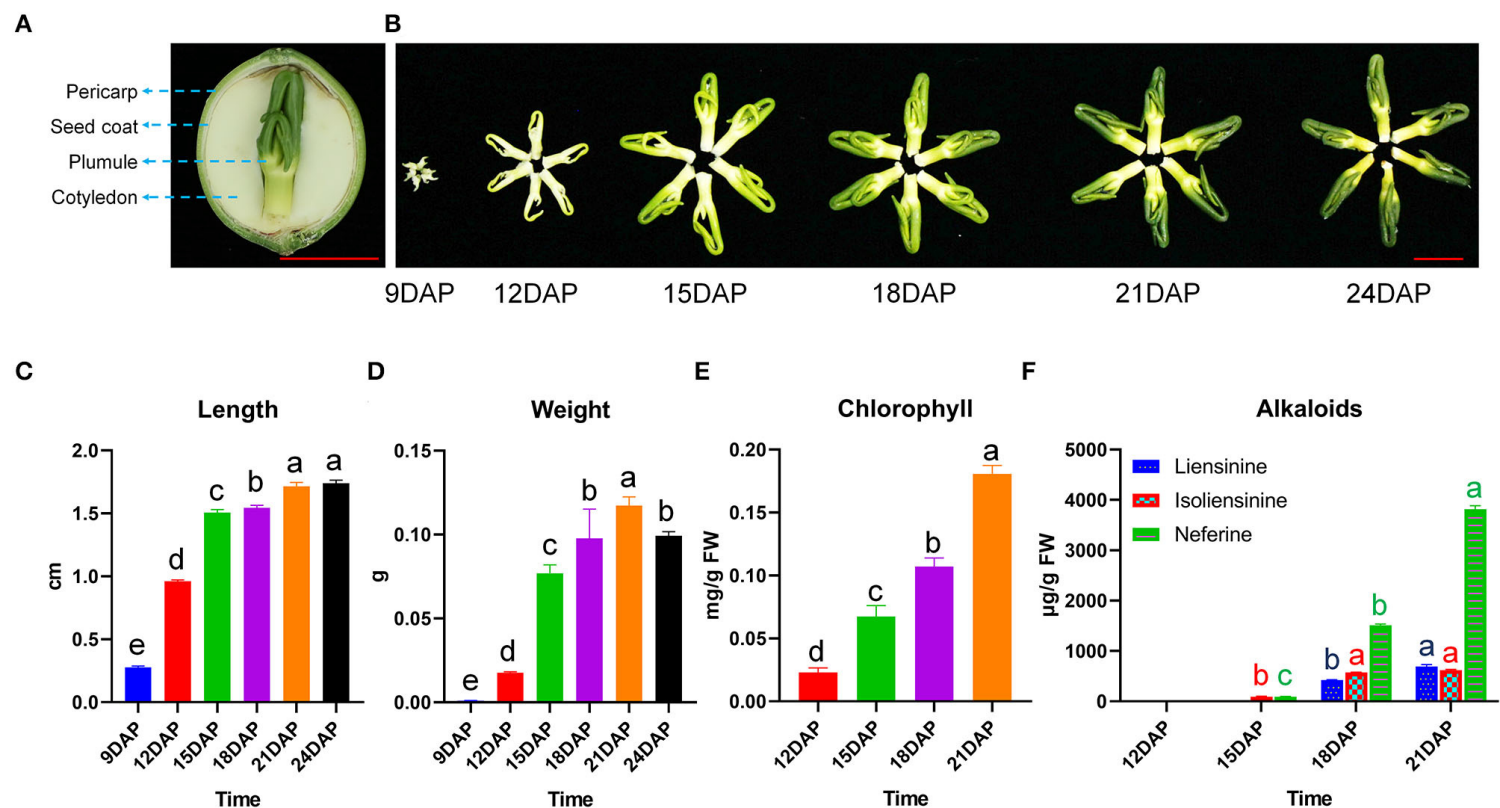
Phenotypic changes during lotus plumule development in cv. “Jianxuan 17”. **(A)** The structure of lotus seed. **(B)** Morphological changes in lotus plumule during development. The red bars represent 1 cm. **(C)** Length, and **(D)** Weight of lotus plumule at different developmental stages. Error bars represent means ± standard error (*n* = 3). **(E)** Chlorophyll, and **(F)** Alkaloid contents in lotus plumule at different developmental stages. Error bars represent means ± standard error (*n* = 4). Statistical significance is based on the Least Significant Difference (LSD) test at *P* < 0.05, one-way ANOVA. Different letters over bars indicate significant difference.

The plumule is located within the lotus seed, enclosed by layers of integuments, including pericarp, seed coat, and cotyledon (Figure 1A). Significant morphological changes in size, weight, and color were observed between 9 and 24 DAP (Figure 1B). For example, the plumule of lotus cv. “Jianxuan 17” (hereafter abbreviated, JNP) showed a rapid increase in length from 9 to 15 DAP, followed by a slow increase to about 1.74 cm at 24 DAP (Figure 1C). JNP weight increased continuously to a highest of 0.117 g at 21 DAP and then decreased to 0.099 g at 24 DAP (Figure 1D). In contrast, the plumule of lotus cv. “China Antique” (hereafter abbreviated, CNP) showed a comparatively smaller size, with a recorded weight of 0.063 g at 21 DAP, which represented a 53.9% decrease in comparison to that of JNP (Supplementary Figures 1A–C). In addition, rapid synthesis and accumulation of Chl were detected from 12 to 21 DAP, which was consistent with the observed change in plumule color (Figure 1E).

The HPLC identification of alkaloid components in lotus plumule showed that JNP mainly accumulated liensinine, isoliensinine, and neferine, with isoliensinine and neferine being detected at 15 DAP but liensinine being detected at 18 DAP (Figure 1F and Supplementary Figure 2). The total alkaloid content in JNP increased from 187.24 μg/g at 15 DAP to 5,130.1 μg/g at 21 DAP, with neferine as the most dominant bis-BIA. In contrast, the total alkaloid content in CNP increased from 67.28 μg/g at 15 DAP to 4,254.6 μg/g at 21 DAP, with liensinine and neferine as the main components detected (Supplementary Figures 1D, 2). These results indicate obvious variation in the plumule alkaloid components of the two tested lotus varieties.

### Transcriptome Profiling of Lotus Plumule During Development

**Figure 2:**
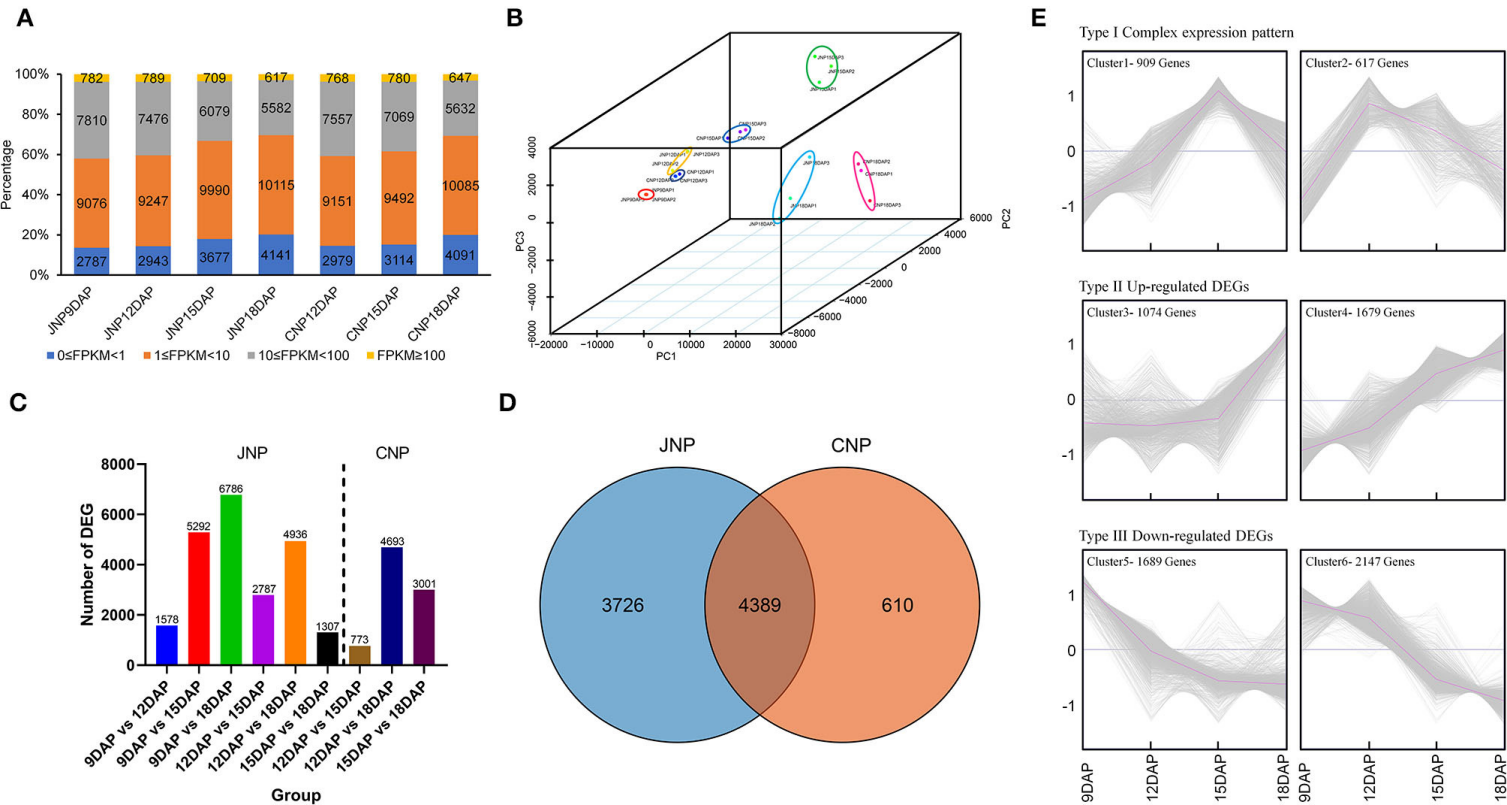
Transcriptome profiling and identification of differentially expressed genes (DEGs) in lotus plumule. **(A)** The gene FPKM expression levels of each sample. **(B)** 3D-PCA plot showing sample clusters based on gene expression levels. **(C)** Number of DEGs between different comparison groups. JNP and CNP represent lotus plumule of cv. “Jianxuan 17” and cv. “China Antique,” respectively. **(D)** Venn diagram showing the number of overlapping DEGs between JNP and CNP. **(E)** Cluster analysis of 8,115 DEGs identified in JPN based on the K-means method. The gray and purple lines in the panels indicate the expression levels of individual genes and the consensus of all genes in a specific subcluster.

To investigate the molecular mechanisms of lotus plumule development, 21 RNA libraries, including JNP at 9, 12, 15, and 18 DAP and CNP at 12, 15, and 18 DAP were constructed. A total of ~588.65 million paired-end clean reads were obtained after conducting quality control of sequencing data. The average GC content was 46.39%, and the average ≥ Q30 (the percentage base which the quality value of clean data is ≥30) of each library was 95.59% (Supplementary Table 3). Approximately 95.56% of the clean reads were mapped to 29,568 genes in the reference genome of “Chinese Antique” (Ming et al., 2013), and 20,455 genes with FPKM expression > 1 in at least one sample were identified. Analysis of the overall distribution of gene expression levels in each sample revealed that FPKM of most genes were in the ranges of 1–10 and 1– 100 (Figure 2A). Principal component analysis (PCA) and correlation coefficient heatmap of samples showed that the three biological replicates were closely clustered (Figure 2B and Supplementary Figure 3).

### Identification of Differentially Expressed Genes in Lotus Plumule

For JNP, a total of 1,578, 5,292, 6,786, 2,787, 4,936, and 1,307 DEGs were identified in the 9_vs._12 DAP, 9_vs._15 DAP, 9_vs._18 DAP, 12_vs._15 DAP, 12_vs._18 DAP, and 15_vs._18 DAP comparison groups, respectively (Figure 2C). Venn diagram showed that the highest number of common DEGs of 4,544 was between 9_vs._15 DAP and 9_vs._18 DAP groups, and the lowest number of common DEGs of 205 was between 9_vs._12 DAP and 15_vs._18 DAP groups (Supplementary Figure 4A). In addition, 100 common DEGs were identified in all the six comparison groups (Supplementary Figure 4A). For CNP, a total of 773, 4,693, and 3,001 DEGs were identified in the 12_vs._15 DAP, 12_vs._18 DAP, and 15_vs._18 DAP comparison groups, respectively (Figure 2C). In addition, 403 common DEGs were identified in all the three groups, with the highest DEG overlap of 2,748 being observed between 12_vs._18 DAP and 15_vs._18 DAP groups (Supplementary Figure 4B). Moreover, 3,726 and 610 DEGs were exclusively identified in JNP and CNP, respectively, while 4,389 common DEGs, accounting for 87.8% of all DEGs in CNP, were identified between the two lotus varieties, suggesting a high similarity in their plumule developmental process (Figure 2D).

### Functional Enrichment of DEGs

The KEGG analysis was used to analyze the functional enrichment of common and exclusive DEGs between JNP and CNP. As a result, 4,389 common DEGs were significantly enriched in 42 KEGG pathways (*P* ≤ 0.05), such as metabolic pathways, biosynthesis of secondary metabolites, carbon fixation in photosynthetic organisms, and photosynthesis (Supplementary Table 4). Specific DEGs from JNP or CNP were shown to be significantly enriched in 19 or 7 pathways, respectively, with those from JNP being involved in metabolic pathways, plant hormone signal transduction, purine metabolism, and arginine biosynthesis, while those from CNP, being involved in fatty acid elongation, fatty acid metabolism, and brassinosteroid biosynthesis (Supplementary Table 4).

A total of 8,115 DEGs identified in JPN were used to investigate the enriched pathways of DEGs with different expression patterns. These DEGs could be classified into three categories based on expression patterns and six clusters using K-means clustering (Figure 2E). In the type I category, 1,526 DEGs showed complex expression patterns, with the highest expression abundance at 15 and 12 DAP in clusters 1 and 2, respectively. In cluster 1, 909 DEGs were enriched in 22 pathways, including metabolic pathways, photosynthesis, and isoquinoline alkaloid biosynthesis (Supplementary Figure 5). Most DEGs in cluster 2 were involved in metabolic pathways, biosynthesis of secondary metabolites, and biosynthesis of amino acids (Supplementary Figure 5). In the type II category, 2,753 DEGs showed an overall upregulated expression pattern, with cluster 4 showing a more continuous upregulated expression than cluster 3 (Figure 2E). Gene functional enrichment analysis indicated that more DEGs in the type II category were enriched in multiple metabolic pathways, such as biosynthesis of secondary metabolites, phenylpropanoid biosynthesis, and carotenoid biosynthesis for cluster 3, and in flavonoid biosynthesis, ubiquinone and another terpenoid-quinone biosynthesis, and starch and sucrose metabolism for cluster 4 (Supplementary Figure 5). In the type III category, 3,836 DEGs showed an overall downregulated expression pattern, with 10 and 29 KEGG pathways being, respectively enriched in clusters 5 and 6, such as metabolic pathways, plant hormone signal transduction, and purine metabolism for cluster 5, and DNA replication, cysteine, and methionine metabolism, and citrate cycle (TCA cycle) for cluster 6 (Figure 2E and Supplementary Figure 5).

### Identification of Key Structural Genes in bis-BIAs Biosynthetic Pathway

The common bis-BIAs biosynthetic pathway is derived from L-tyrosine metabolism (Hagel and Facchini, 2013). Thus, we initially analyzed the expression patterns of key genes involved in the tyrosine biosynthetic pathway (Supplementary Figure 6). As a result, upregulated expression of a key enzyme of the glycolysis pathway, *NnPFK* (NNU_10589), encoding ATP-dependent 6-phosphofructokinase, and a key regulatory enzyme of the pentose phosphate pathway (PPP), *NnG6PD* (NNU_02159), encoding glucose 6-phosphate dehydrogenase were observed during lotus plumule development (Supplementary Figure 6B). Similarly, upregulated expression of *NnSK* (NNU_20134), *NnCS* (NNU_13158), and *NnADH* (NNU_08507), encoding shikimate kinase, chorismate synthase, and arogenate dehydrogenase, respectively, were observed. In contrast, *NnDHD-SDH* (NNU_06891) and *NnPPA-AT* (NNU_20211), encoding bifunctional 3-dehydrogenate dehydratase/shikimate dehydrogenase and prephenate aminotransferase, respectively, were downregulated between 12 and 15 DAP. Notably, *NnCM* (NNU_04572) encoding chorismate mutase, which catalyzes the first committed step in the assembly of tyrosine, showed the highest expression abundance at 12 and 15 DAP in JNP and CNP (Supplementary Figure 6B).

**Figure 3:**
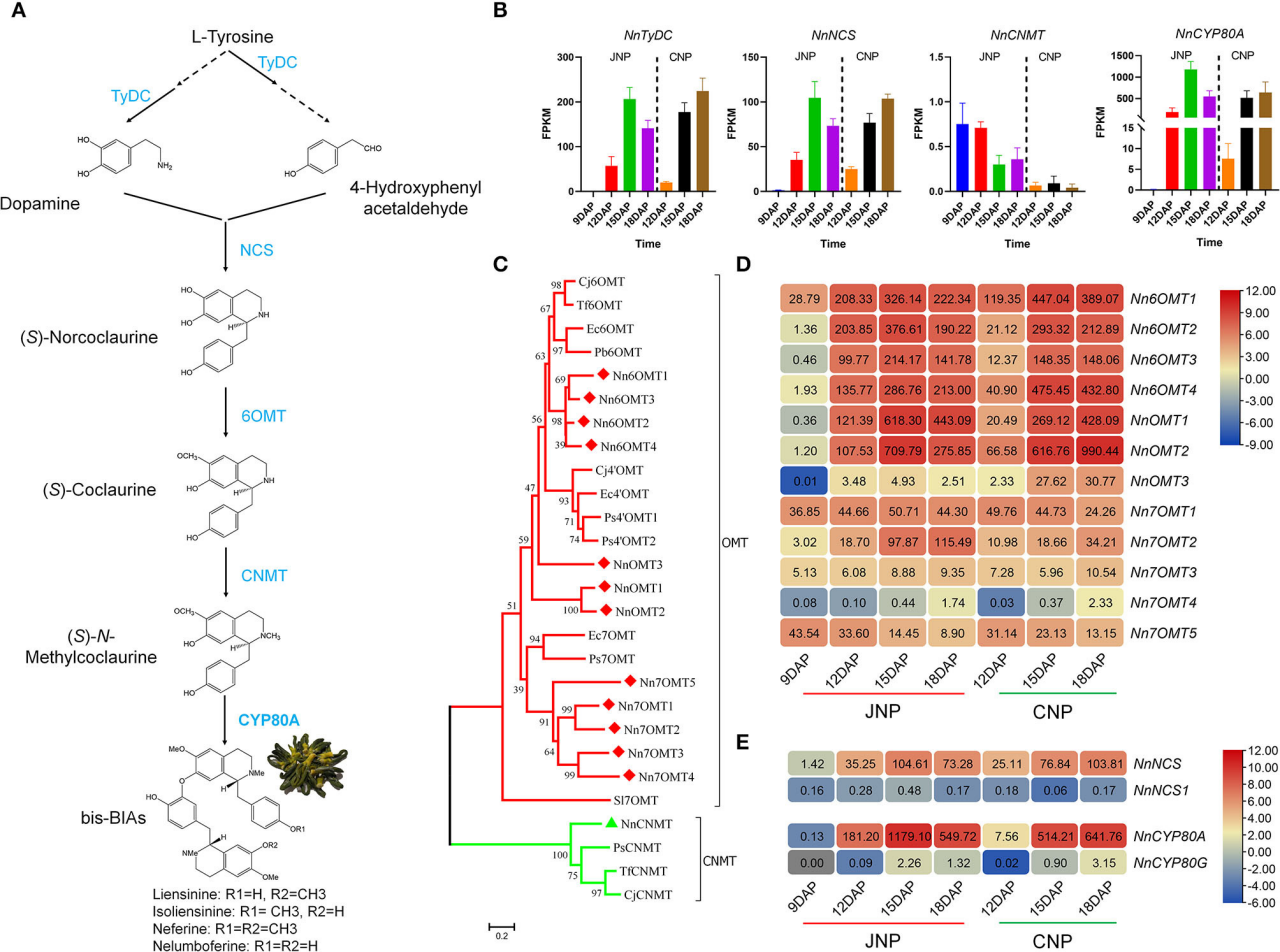
Identification and expression analysis of bis-BIAs biosynthesis genes. **(A)** Key steps in the predicted bis-BIAs biosynthesis pathway in lotus plumule. **(B)** Expression patterns of *NnTyDC* (NNU_22559), *NnNCS* (NNU_14334), *NnCNMT* (NNU_11880), and *NnCYP80A* (NNU_21373) during lotus plumule development. **(C)** Phylogenetic tree depicting the relationship between differentially expressed *O*-methyltransferase (OMT) genes. GenBank Accession Number: Cj6OMT (BAB08004.1), Tf6OMT (ACO90238.1), Ec6OMT (BAM37634.1), Pb6OMT (ACO90225.1), Cj4'OMT (Q9LEL5.1), Ec4'OMT (BAM37633.1), Ps4'OMT1 (XP_026430708.1), Ps4'OMT2 (XP_026440860.1), Ec7OMT (BAE79723.1), Ps7OMT (XP_026440002), PsCNMT (Q7XB08.1), TfCNMT (Q5C9L6.1), and CjCNMT (BAB71802.1). **(D)** Expression patterns of OMT genes during lotus plumule development. **(E)** A heatmap showing expression differences between *NnNCS* and *NnCYP80* homolog members.

The putatively common BIAs biosynthetic pathway in the lotus plumule is shown in Figure 3A. DEGs associated with key lotus BIAs' biosynthetic pathways such as, *NnTyDC* (NNU_22559), *NnNCS* (NNU_14334), and *NnCYP80A* (NNU_21373) encoding TyDC, NCS, and CYP80A, respectively, were highly expressed in the plumule (Figure 3B). Phylogenetic analysis was performed to determine the evolutionary relationships with their homologous genes from other plant species (Supplementary Figure 7A). *NnTyDC, NnNCS*, and *NnCYP80A* were initially upregulated from 9 to 15 DAP and then downregulated at 18 DAP in JNP, whereas they were continuously upregulated until 18 DAP in CNP (Figure 3B). Notably, only a single gene copy of *NnCNMT* (NNU_11880) encoding CNMT was identified in the lotus genome with extremely low FPKM expression < 1 in the plumule (Figure 3B).

The *O*-methylation is a crucial step that catalyzes the *O*-methyltransferase (OMT) transfer of a methyl group to a hydroxyl group of an alkaloid substrate leading to the structural diversity of lotus BIAs (Morris and Facchini, 2019; Menendez-Perdomo and Facchini, 2020). Analysis of the structural formula showed that bis-BIAs were *O*-methylated at C6, C7, and C4′, suggesting that 6OMT, 7OMT, and 4′OMT could be involved in the biosynthesis of bis-BIAs in lotus plumule. Twelve differentially expressed OMT genes were identified in the lotus by homology alignment and confirmed by phylogenetic analysis (Figure 3C). Of these, four OMTs, the Nn6OMT1 (NNU_19035), Nn6OMT2 (NNU_23168), Nn6OMT3 (NNU_03166), and Nn6OMT4 (NNU_03165), were closely paired with the 6OMT genes from *Coptis japonica, Thalictrum flavum, Eschscholzia californica*, and *Papaver bracteatum*, respectively. Similarly, five OMTs, including Nn7OMT1 (NNU_04966), Nn7OMT2 (NNU_04906), Nn7OMT3 (NNU_20903), Nn7OMT4 (NNU_20253), and Nn7OMT5 (NNU_16993), were closely clustered with 7OMT gene clusters from *Eschscholzia californica* and *Papaver somniferum*. The remaining three differentially expressed OMTs, including NnOMT1 (NNU_15801), NnOMT2 (NNU_15809), and NnOMT3 (NNU_25948), were both clustered with 6OMT and 4'OMT genes. Gene expression analysis showed that all differentially expressed OMTs except *Nn7OMT5* were upregulated from 9 to 15 DAP in JNP, with *Nn7OMT5* showing a continuously downregulated expression from 12 to 18 DAP in both JNP and CNP (Figure 3D). In addition, *NnOMT3* and *Nn7OMT2* genes exhibited obvious expression differences between JNP and CNP, with *NnOMT3* having a 12.3-fold increase in expression abundance in CNP than in JNP at 18 DAP (Figure 3D). Similarly, a 3.4-fold increase in the expression abundance of *Nn7OMT2* (NNU_04906) was observed in JNP at 18 DAP.

Using five BIAs' biosynthesis-related genes, the qRT-PCR analysis, including *NnTyDC, Nn6OMT1, Nn6OMT2, Nn6OMT3*, and *NnCYP80A*, was further conducted to validate the RNA-Seq data. As a result, a higher correlation between RNA-Seq data and qRT-PCR results was observed, suggesting strong RNA-Seq data reliability in this study (Supplementary Figure 7B). In addition, the determination of expression levels of bis-BIAs biosynthesis genes in other lotus tissues, including leaf, petiole, rhizome, and root using the publicly available transcriptome data (Shi et al., 2020), revealed that most genes were highly expressed in leaf (Supplementary Figure 8). Interestingly, highly expressed genes in leaf tissues included *Nn6OMT2, NnCYP80A*, and *NnOMT1*, thus, suggesting that some bis-BIAs biosynthesis genes could also be involved in the biosynthesis of aporphine-type BIAs in lotus leaves.

### BIAs' Biosynthesis Gene Pairs Show Functional Redundancy and Divergence Between Paralogous Members

Gene mapping analysis identified 16 bis-BIAs biosynthesis genes distributed across six lotus chromosomes (Chr), with seven genes localized on Chr1 (Supplementary Figure 9A). Notably, possible gene duplication events in some OMTs were observed. For example, *NnOMT1* and *NnOMT2* genes located in a 42.2 kb region on Chr1 shared about 83.4% amino acid sequence identity (Supplementary Figure 9B). Similarly, *Nn6OMT2, Nn6OMT3*, and *Nn6OMT4* genes located in a 354.7kb interval on Chr1 shared about 79.6% identity (Supplementary Figure 9C). The observed high transcript abundance of these OMT genes during plumule development suggested their functional redundancy and their synergistic interactions to accumulate bis-BIAs in lotus.

In addition, paralogs of two structural genes involved in alkaloid synthesis were identified, suggesting their likely functional divergence in lotus. For example, *NnNCS1* (NNU_21731) and its *NnNCS* homolog shared about 59.3% amino acid sequence identity (Supplementary Figure 9D). However, the expression of *NnNCS1* was extremely low in the lotus plumule (Figure 3E). Moreover, *NnCYP80A* and its homolog *NnCYP80G* (NNU_21372) located within a 26.1-kb interval shared about 59.18% identity, with the latter showing an extremely low expression level in lotus plumule (Figure 3E and Supplementary Figure 9E). Overall, these results suggest a degree of functional specialization between the paralogs of *NnNCS* and *NnCYP80* genes.

### Genome-Wide Identification of Chl Biosynthesis Genes in Lotus

**Figure 4:**
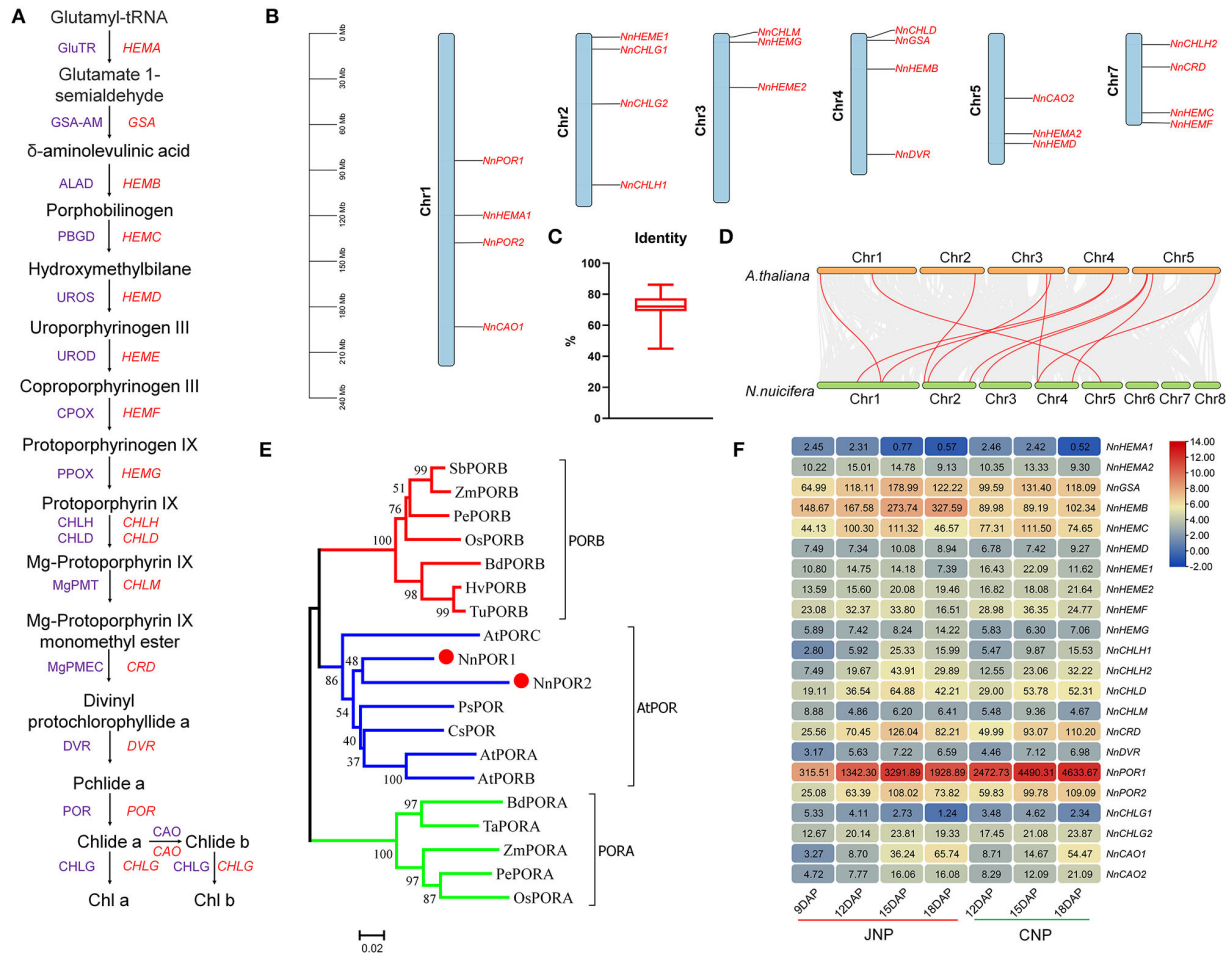
Genome-wide identification and expression analysis of Chl biosynthesis genes in lotus. **(A)** Chl biosynthesis pathway in higher plants. Purple and red letters represent enzymes and genes, respectively. **(B)** Chromosomal localization of Chl biosynthesis genes in lotus. **(C)** The amino acid sequence identity between *Arabidopsis* and lotus Chl biosynthesis genes. **(D)** Synteny analysis of Chl synthesis genes between *Arabidopsis* and lotus. **(E)** Phylogenetic tree showing the relationship of lotus POR genes. GenBank Accession Number: SbPORB (XP_002467010.1), ZmPORB (NP_001149903.1), PePORB (PH01000789G0260), OsPORB (Q8W3D9.1), BdPORB (XP_003570527.1), HvPORB (Q42850.1), TuPORB (TuPORB), AtPORC (NP_001030948.1), PsPOR (CAA44786.1), CsPOR (BAA21089.1), AtPORA (AAC49043.1), AtPORB (NP_001031731.1), BdPORA (XP_010240614.1), TaPORA (Q41578.1), ZmPORA (NP_001167683.1), PePORA (PH01000087G0190), and OsPORA (Q7XKF3.1). **(F)** Expression patterns of Chl biosynthesis genes during lotus plumule development.

The Chl biosynthesis is crucial for lotus plumule development, and the process was accompanied by changes in color from light yellow at 9 DAP to dark green at 18 DAP (Figures 1B,E). Twenty-two genes encoding 16 key enzymes in the Chl biosynthesis pathway were found distributed across six chromosomes in the lotus genome (Figures 4A,B). The average amino acid sequence identity between the lotus and *Arabidopsis* homologous Chl genes was 71.6% (Figure 4C). In addition, collinearity analysis between lotus and *Arabidopsis* genes identified 11 colinear gene pairs, encoding GSA-AM (*NnGSA*, NNU_22236), UROD (*NnHEME1*, NNU_12265), CHLG (*NnCHLG1*, NNU_12622), POR (*NnPOR1*, NNU_01188; *NnPOR2*, NNU_16195), CHLH (*NnCHLH1*, NNU_03121), PPOX (*NnHEMG*, NNU_02121), DVR (*NnDVR*, NNU_06919), and CAO (*NnCAO2*, NNU_24327), which suggested that the Chl biosynthesis pathway is conserved between the two plants (Figure 4D).

The Pchlide reduction is catalyzed by pchlide oxidoreductase (POR) and presents the penultimate step in the Chl biosynthesis pathway. Here, two genes, *NnPOR1* and *NnPOR2*, encoding light-dependent Pchilde oxidoreductase (LPOR) were identified in the lotus, and phylogenetic analysis showed their close pairing with *Arabidopsis* POR genes (Figure 4E). In addition, multiple sequence alignments revealed that all *NnPOR* genes contained a conserved NADPH-binding motif, TGASSGLG, and an active YKDSK site motif (Supplementary Figure 10).

The expression patterns of lotus Chl biosynthesis genes were similar between JNP and CNP, with 20 genes showing differential expression profiles during plumule development (Figure 4F). Thirteen genes exhibited upregulated profiles from 9 to 15 DAP, which was later downregulated at 18 DAP, such as *NnGSA, NnHEMC* (NNU_01206), and *NnCRD* (NNU_10837). Four genes, including *NnHEMB* (NNU_04375), *NnHEMG*, and *NnCAO* (NNU_24327, NNU_19596) were continuously upregulated throughout the tested lotus plumule developmental stages (Figure 4F). Using six Chl biosynthesis genes, the qRT-PCR analysis, including *NnPOR1, NnPOR2, NnCAO1 NnCHLG2, NnHEMB*, and *NnCHLD*, revealed a higher correlation between RNA-Seq data and qRT-PCR results at 9, 12, and 15 DAP (Supplementary Figure 11A). Tissue expression analysis showed that most Chl biosynthesis genes were preferentially expressed in lotus leaf, while *NnHEMF* (NNU_00797) and *NnHEMG* were highly expressed in non-photosynthetic tissues (Supplementary Figure 11B).

### Activated Expression of Photosynthesis-Related Genes During Lotus Plumule Development

**Figure 5:**
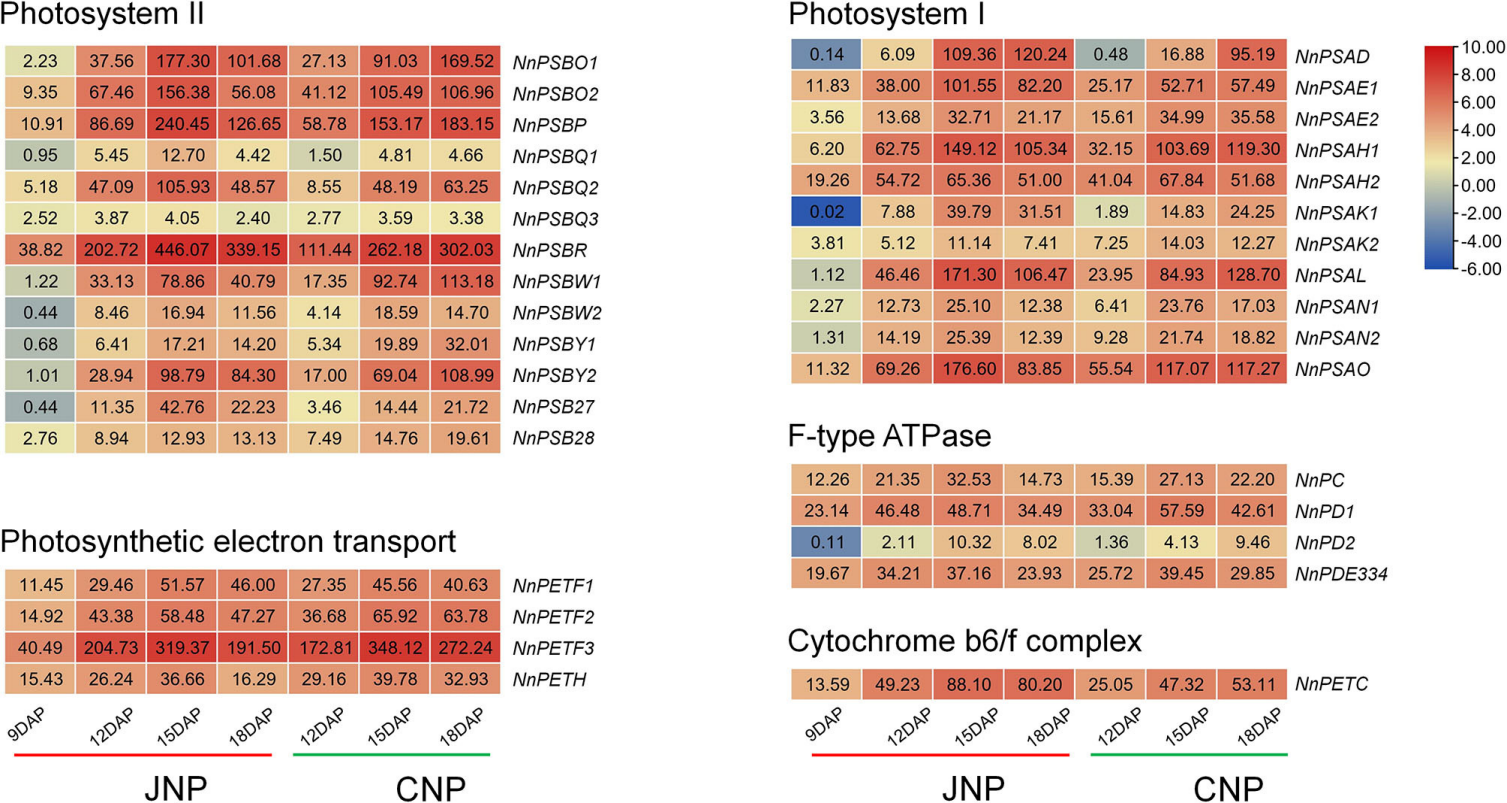
Expression analysis of photosynthesis-related genes during lotus plumule development.

Photosynthesis in green plants is the process of transforming light energy into chemical energy. With the biosynthesis of Chl, the photosynthesis pathway was activated during lotus plumule development in JNP and CNP (Figure 5 and Supplementary Figure 12). Forty-eight differentially expressed photosynthesis-related genes were detected, including 15 genes involved in light-harvesting chlorophyll-protein complex, 11 Photosystem I genes, 13 Photosystem II genes, four photosynthetic electron transport genes, four F-type ATPase genes, and one Cytochrome b6/f complex gene. Notably, these genes exhibited continuous upregulated profiles from 9 to 15 DAP in JNP, such as photosystem I subunit VI *NnPSAH* (NNU_21431, NNU_26150), photosystem II oxygen-evolving enhancer protein *NnPSBO* (NNU_05490, NNU_23333), and ferredoxin *NnPETF* (NNU_05621, NNU_06707, and NNU_09587). The upregulated levels of these genes were consistent with the expression patterns of most Chl biosynthesis genes (Figure 5 and Supplementary Figure 12). These results indicated a simultaneous activation of photosynthesis and Chl biosynthesis pathways during lotus plumule development.

### Chl Is Synthesized by the Light-Dependent Reaction in Lotus Plumule

**Figure 6:**
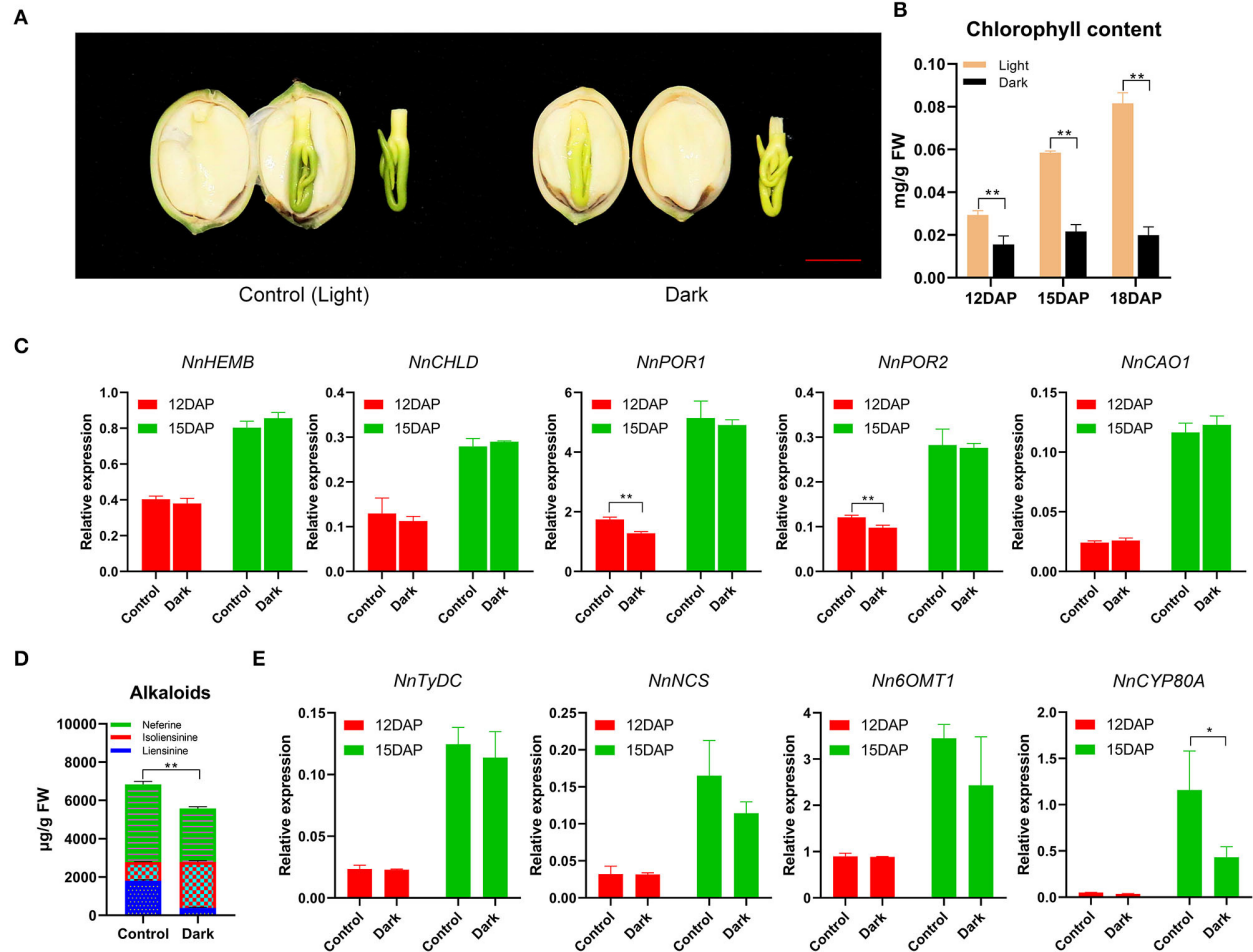
The biosynthesis of Chl and alkaloids in lotus plumule is inhibited under dark treatment. **(A)** Phenotypes of 18 DAP lotus plumule under control (left) and dark treatments (right). The red bar represents 1 cm. **(B)** Chl content in lotus plumule under light and dark treatments. Bars represent means ± standard error (*n* = 4). **P ≤ 0.01. **(C)** Expression patterns of Chl biosynthesis genes under light and dark conditions. Bars represent means ± standard error (*n* = 3). **P ≤ 0.01. **(D)** Alkaloid content in lotus plumule under light and dark conditions. Bars represent means ± standard error (*n* = 4). **P ≤ 0.01. **(E)** Expression patterns of selected alkaloid biosynthesis genes under light and dark conditions. Bars represent means ± standard error (*n* = 3). *P ≤ 0.05.

To investigate the relationship between light and Chl biosynthesis in lotus plumule, a light-controlled experiment was performed in the pods of seed-lotus cultivar “Jianxuan17”. As a result, Chl biosynthesis in lotus plumule was strongly inhibited under the dark condition with plumule color turning yellowish, and Chl content decreased by 75.5%, relative to the unwrapped pods at 18 DAP (Figures 6A,B). In gymnosperms, algae, and photosynthetic bacteria, light-independent Pchilde reductase (DPOR) is responsible for Chl biosynthesis in dark conditions. Screening for homologous DPOR genes using the published lotus chloroplast (Wu et al., 2014) and nuclear (Ming et al., 2013) genome data revealed no hits (Supplementary Figure 13). In contrast, two LPOR genes were significantly upregulated from 9 to 15 DAP. For example, the FPKM expression of *NnPOR1* showed a significant increase from 351.51 at 9 DAP to 3291.89 at 15 DAP (Figure 4F). Overall, these results demonstrate that Chl biosynthesis in lotus plumule is light-dependent, catalyzed by the LPOR reduction of Pchlide. Notably, plumule exposure to dark conditions had no significant effect on the expression of Chl biosynthesis genes. For example, no variation in the expression of five Chl biosynthesis genes, including *NnHEMB, NnCHLD, NnPOR1, NnPOR2*, and *NnCAO1*, was observed in samples under light and dark treatments at 12 and 15 DAP (Figure 6C).

Light is a key factor affecting the biosynthesis of secondary metabolites (Coelho et al., 2007; Setiawati et al., 2018). The content of bis-BIAs in lotus plumule under dark treatment decreased by 18.4% relative to those under normal light exposure at 18 DAP, which was consistent with the decrease in the expression of BIAs pathway genes, such as *NnNCS, Nn6OMT*, and *NnCYP80A* (Figures 6D,E).

### Identification of Transcription Factors Co-Expressed With bis-BIAs and Chl Biosynthesis Genes

**Figure 7:**
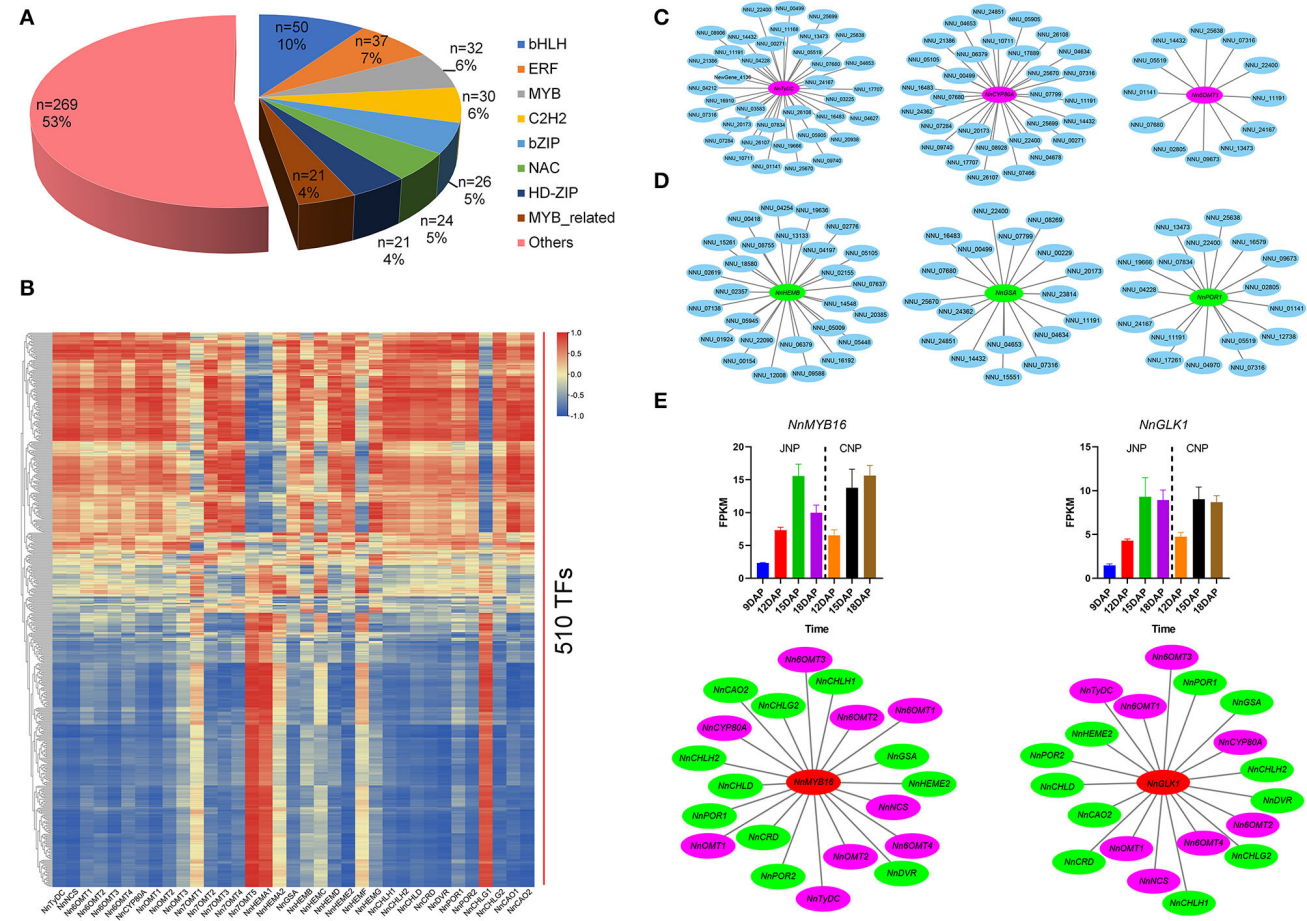
Identification of transcription factors co-expressed with bis-BIAs and Chl biosynthesis genes. **(A)** The proportion of differentially expressed TFs. **(B)** Visual representation of the co-expression relationships between 510 TFs and bis-BIAs/ Chl biosynthesis genes. **(C)** Selected bis-BIAs, and **(D)** Chl biosynthesis genes co-expressed with TFs. **(E)** The FPKM expression of *NnMYB16* (NNU_07316) and *NnGLK1* (NNU_11191), and their co-expression with multiple bis-BIAs and Chl biosynthesis genes, respectively.

Transcription factors (TFs) are master regulators of gene expression (Mitsis et al., 2020). A total of 510 differently expressed TFs from 50 TF families were identified in this study, with the majority being bHLH, ERF, MYB, and C2H2 TF family genes (Figure 7A). Varied expression patterns were observed among these TFs, for example, of the 50 bHLH TFs identified, 11 were continuously upregulated, and 16 were downregulated, whereas the remaining 23 had irregular expression patterns (Supplementary Figure 14A). Functional enrichment analysis of the 510 TFs showed that plant hormone signal transduction and DNA-binding transcription factor activity were the most enriched KEGG and GO terms, respectively. In addition, “response to chitin” and “cell differentiation” were the most enriched terms in biological process classification, and genes involved in these two processes showed varied expression patterns (Supplementary Figure 14B).

To investigate the potential functions of TFs involved in bis-BIAs and Chl biosynthesis, the correlation between their expression profiles and of the identified bis-BIAs and Chl biosynthesis structural genes was calculated (Figure 7B and Supplementary Table 5). For example, 37, 30, and 12 TFs were co-expressed with *NnTyDC, NnCYP80A*, and *Nn6OMT1*, while, 27, 18, and 17 TFs were co-expressed with *NnHEMB, NnGSA*, and *NnPOR1* (*r* ≥0.8), respectively (Figures 7C,D). Interestingly, TFs, such as *NnMYB16* (NNU_07316) and *NnGLK1* (NNU_11191), showed co-expression with multiple structural genes (Figure 7E). MYB TFs are key regulators of plant secondary metabolite biosynthesis (Chezem et al., 2017; Kishi-Kaboshi et al., 2018). A candidate *NnMYB16* gene showed a continuous upregulated expression from 9 to 15 DAP, which was subsequently downregulated at 18 DAP in JNP. In addition, *NnMYB16* was co-expressed with nine bis-BIAs biosynthetic genes and 11 Chl biosynthetic genes. Similarly, GARP-type GLK TFs are key regulators of Chl biosynthesis, and *NnGLK1*, a homolog of *AtGLK1* (AT2G20570), has been shown to bind to the promoter of some Chl biosynthetic genes and regulate their expression (Waters et al., 2009; Sakuraba et al., 2017). In this study, a correlation was observed between the expression of *NnGLK1* and 11 Chl biosynthetic genes, including *NnGSA, NnCHLD*, and *NnPOR2* during lotus plumule development. In addition, *NnGLK1* also was co-expressed with eight bis-BIAs biosynthetic genes. Overall, these results provide important references for further research on the transcriptional regulation mechanisms of bis-BIAs and Chl biosynthesis in the lotus.

## Discussion

Recent studies on the development of lotus seeds have mainly focused on cotyledons, the main edible seed tissue (Wang et al., 2016; Li et al., 2018; Sun et al., 2020). However, despite the pharmacological significance of lotus plumule, its development process remains largely unknown. Physiological analysis in this study determined the rapid growth stage of lotus plumule to be from 9 to 15 DAP, while the onset and rapid accumulation of BIAs biosynthesis were detected at 15 DAP (Figure 1). The observed variation in plumule color during development occurred due to biosynthesis of Chl, which was rapidly accumulated between 12 and 18 DAP.

### Dynamic Characteristics of BIAs Biosynthesis in Lotus

Most parts of the lotus plant have traditionally been used for various medicinal purposes due to its ability to accumulate abundant bioactive compounds, such as alkaloids and flavonoids (Mukherjee et al., 2009; Chen et al., 2012; Deng et al., 2016; Limwachiranon et al., 2018). To date, over 20 alkaloids categorized into aporphines, monobenzylisoquinolines, and bisbenzylisoquinolines have been identified in lotus (Deng et al., 2016; Yang et al., 2017). In this study, bis-BIAs, such as liensinine, isoliensinine, and neferine, were identified as the predominant alkaloids in lotus plumule, which is consistent with the results of previous studies (Deng et al., 2016; Menendez-Perdomo and Facchini, 2020). Notably, isoliensinine was not detected in CNP, which suggests the effects of genotype on alkaloid composition in lotus plumule (Figure 1F and Supplementary Figure 1D). In contrast, aporphine-type BIAs, including *N*-nornuciferine, *O*-nornuciferine, anonaine, nuciferine, and romaine, were predominantly accumulated in the lotus leaf (Chen et al., 2013; Deng et al., 2016). This observed abundant accumulation of different alkaloid types underscores the numerous pharmacological potentials of the lotus plant.

The biosynthesis pathway of aporphine-type BIAs in lotus leaf has previously been reported (Yang et al., 2017; Deng et al., 2018). However, the bis-BIAs biosynthesis pathway in plumule is yet to be characterized. Here, 16 structural genes potentially involved in bis-BIAs biosynthesis, including *NnTyDC, NnNCS, Nn6OMT*, and *NnCYP80A*, were identified in the lotus plumule (Figure 3). The accumulation of bis-BIAs was detected from 15 DAP, while most related biosynthesis genes were significantly upregulated from 12 DAP, and this suggests a delayed initiation of bis-BIAs biosynthesis after structural gene activation in lotus plumule (Figures 3B,D). Decarboxylation of tyrosine to yield *N*-methylcoclaurine is the most common pathway of alkaloids biosynthesis (Hagel and Facchini, 2013). However, our results identified genes with contrasting expression patterns in both bis-BIAs and aporphine-type BIAs biosynthesis. For example, the expression of *Nn6OMT1* (NNU_19035) was downregulated in aporphine-type BIAs biosynthesis in leaf (Yang et al., 2017) but was significantly upregulated during bis-BIAs biosynthesis in plumule (Figure 3D). Similarly, *NnCYP80G*, a homolog of *NnCYP80A* and a potential structural gene in the aporphine-type BIAs biosynthesis (Deng et al., 2018), was highly expressed in lotus leaf but with extremely low expression levels in the plumule (Figure 3E). Taken together, these results suggest flexibility in the lotus alkaloids biosynthesis.

*NnCNMT* is a single gene copy in the lotus genome that encodes the CNMT enzyme, which catalyzes the conversion of (*S*)-Coclaurine to (*S*)-*N*-Methylcoclaurine. The expression of *NnCNMT* was upregulated in the lotus leaf (Yang et al., 2017) but was barely detectable in the plumule (Figure 3B). This result contradicts the independent production of alkaloids in lotus plumule, thus additional proteomic and enzyme activity studies on CNMT are warranted. Interestingly, previous identification of BIAs in leaf bleeding sap led to the speculation that bis-BIAs are mainly synthesized in the leaf and then transported to the plumule (Deng et al., 2016); thus, our results provide additional evidence supporting this bis-BIAs accumulation pattern in lotus plumule.

### Characterization of Chl Biosynthesis in Lotus Plumule

The Chl biosynthesis is an essential cellular process for plant photosynthesis. Unlike most crops, the lotus plumule is green in color due to the presence of Chl in its seeds, which is an adaptive trait for seeds' vitality and longevity (Ji et al., 2001; Shen-Miller, 2007). All structural genes related to the Chl biosynthesis pathway and their homologs were identified in the lotus genome, with the expression levels of most genes showing a positive correlation with Chl content in the plumule (Figure 4). This result suggests that the Chl biosynthesis pathway is conserved in lotus. However, a comparison between lotus and *Arabidopsis* Chl biosynthesis pathway-related genes identified some independent evolutionary patterns. For example, a different number of isoforms of HEMA, GSA, POR, and CAO were observed, with *Arabidopsis* having three, two, three, and one while lotus having two, one, two, and two isoforms, respectively. *NnCHLG1* and its homolog *NnCHLG2*, encoding Chl synthase and catalyzing the last step of Chl biosynthesis, showed contrasting expression patterns during lotus plumule development (Figure 4F). Similarly, inconsistent expression patterns were also observed in *NnHEMA1* and *NnHEMA2* genes (Figure 4F). The contrasting expression patterns between the paralogs of *CHLG* and *HEMA* could suggest that the genes are undergoing functional divergence in the lotus. Furthermore, the expression levels of *NnHEMD* and *NnCHLM*, which are single-copy genes encoding uroporphyrinogen III synthase (UROS) and SAM Mg-protoporphyrin IX methyltransferase (MgPMT), respectively, were very low, and their functions in Chl synthesis need to be further determined (Figure 4F).

The green lotus plumule is enclosed in the middle of the seed, and thus could be assumed to undergo light-independent Chl biosynthesis (Yakovlev and Zhukova, 1980). However, previous studies have reported light-dependent Chl biosynthesis in the lotus plumule via specialized chloroplast with giant granum and photosystem structures (Zuo Bao-yu et al., 1992; Ji et al., 2001). In this study, lotus plumule incubated in dark conditions developed yellowish color with severely decreased Chl content, which further confirmed the light-dependent Chl biosynthesis reaction (Figures 6A,B). It is reasonable to speculate that lotus seeds utilize the thin semi-transparent integuments around the plumule during the early stages of development to sense light signals for light-dependent Chl biosynthesis reaction (Ji et al., 2001). In addition, previous anatomical studies identified three pores inside lotus seeds and showed that the tissue structures at both ends of seeds are relatively loose, which could allow light penetration (Chen and Zhang, 1988; Huang et al., 2011). Overall, these results provide potential evidence that the lotus plumule is sensitive to light stimuli at the structural level. Moreover, the absence of genes encoding DPOR in chloroplast and nuclear genome of the lotus was consistent with the previous conclusion that members of DPOR genes were completely lost in angiosperms, thus, inhibiting their ability to form Chl under light-independent reactions (Gabruk and Mysliwa-Kurdziel, 2020). Interestingly, two highly homologous genes encoding LPOR were identified in the lotus genome with significant upregulated expression levels in plumule during Chl biosynthesis (Figures 4E,F), further providing evidence for light-dependent Chl biosynthesis in lotus plumule.

### The Potential Connections Between the Pathways Leading to bis-BIAs and Chl Biosynthesis in Lotus Plumule

The currently available literature has not been able to resolve the connection between BIAs and Chl biosynthesis in plants (Baldwin, 1988; Wei et al., 2012; Setiawati et al., 2018). The correlation between alkaloids and Chl biosynthesis varies among plant species, for example, no significant correlation was observed between purine alkaloids and Chl in green tea cultivars, while a strong correlation existed between alkaloids and Chl biosynthesis in *Ephedra procera* (Parsaeimehr et al., 2010; Wei et al., 2012). In this study, potential connections between these two pathways were detected in the lotus plumule. First, bis-BIAs and Chl showed similar biosynthesis and accumulation patterns in lotus plumule, with both showing continuous accumulation from 15 DAP to 21 DAP, despite a delayed initiation of bis-BIAs biosynthesis relative to Chl biosynthesis (Figures 1E,F). Second, some structural genes of the bis-BIAs and Chl biosynthesis pathways showed similar expression patterns during lotus plumule development. Correlating in the expression of structural genes related to these two synthetic pathways revealed 13 highly co-expressed associations (r ≥ 0.8) between bis-BIAs biosynthesis genes with at least one Chl biosynthesis gene (Supplementary Figure 15). In addition, some co-expressed TFs with bis-BIAs and Chl biosynthesis genes, such as *NnMYB16* and *NnGLK1*, were identified (Figure 7E). As a key Chl biosynthesis regulator, *NnGLK1* also showed co-expressed with some bis-BIAs biosynthesis genes, including *NnNCS, Nn6OMTs*, and *NnCYP80A* (Figure 7E). We therefore speculated that a common transcriptional regulatory mechanism might exist between these two pathways. Third, light is a co-regulator of bis-BIAs and Chl biosynthesis in lotus plumule, and our results showed that bis-BIAs and Chl biosynthesis in lotus plumule were strongly inhibited under dark conditions, with the content of bis-BIAs and Chl decreasing by 18.4 and 75.5% at 18 DAP, respectively, relative under normal light exposure (Figures 6B,D). The light-induced co-regulation of alkaloids and Chl biosynthesis has previously been reported (Zhao et al., 2001; Zhu et al., 2015; Yu et al., 2018; Li et al., 2021). For example, the light improved alkaloids and Chl biosynthesis in the *Catharanthus roseus* callus and enhanced the content of vindoline and Chl in illuminated callus by ~ 3–4 folds and 10–20 folds, respectively (Zhao et al., 2001).

This study provides unprecedented information and resources which could potentially be applied in lotus seed preservation and bis-BIAs detection in the lotus plumule. For example, the flavor quality of fresh lotus seeds deteriorates rapidly from 15 DAP due to increased alkaloid accumulation, leading to bitterness (Tu et al., 2020; Sun et al., 2021). Decreased alkaloid accumulation in lotus plumule under dark treatment was observed in this study. Thus, storing harvested seedpods in the dark could be a practical way to extend the shelf-life of fresh lotus seeds during postharvest storage. In addition, the correlation between bis-BIAs and Chl contents observed in our study suggests that determining Chl content alone could adequately be used as a potentially cost-effective indicator for predicting bis-BIAs content, which is usually expensive and labor-intensive.

## Data Availability Statement

The datasets presented in this study can be found in online repositories. The names of the repository/repositories and accession number(s) can be found at: https://www.ncbi.nlm.nih.gov/, PRJNA747903.

## Author Contributions

MY, YL, and HSu contributed to the conception and design of the study. HSu, HSo, DY, MZ, and YW performed the experiments and data analysis. HSu wrote the manuscript. MY, YL, HSu, XD, JL, JX, and LC revised the manuscript. All authors read and approved the final version of the manuscript.

## Funding

This work was supported by the Biological Resources Program CAS (Grant No. KFJ-BRP-007-009), the National Natural Science Foundation of China (Grant No. 31872136), and the Hubei Provincial Natural Science Foundation of China (Grant No. 2020CFB484).

## Conflict of Interest

The authors declare that the research was conducted in the absence of any commercial or financial relationships that could be construed as a potential conflict of interest.

## Publisher's Note

All claims expressed in this article are solely those of the authors and do not necessarily represent those of their affiliated organizations, or those of the publisher, the editors and the reviewers. Any product that may be evaluated in this article, or claim that may be made by its manufacturer, is not guaranteed or endorsed by the publisher.
