# Supplementary material for: Transcriptome-Wide Characterization of Alkaloids and Chlorophyll Biosynthesis in Lotus Plumule
Source: Front Plant Sci. 2022 May 23;13:885503. doi: 10.3389/fpls.2022.885503 (PMC9168470; doi:10.3389/fpls.2022.885503)
Supplement: Supplementary file 6 [file DataSheet1.pdf]

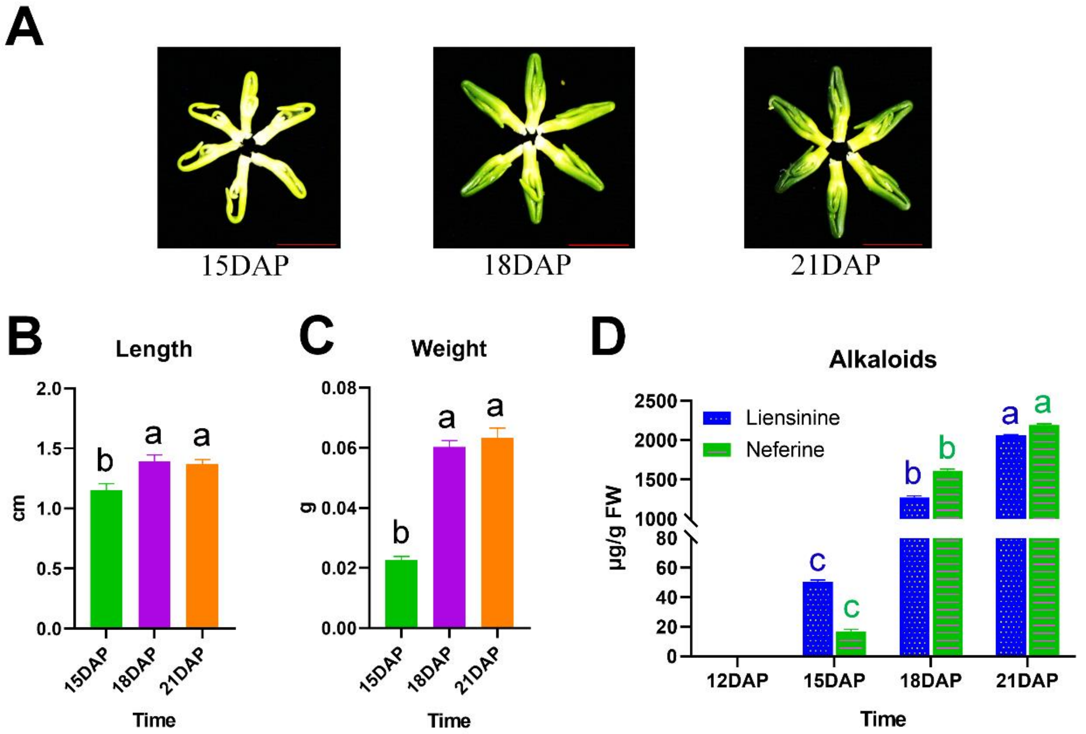

Supplementary Figure 1 The development process of lotus plumule in cv. 'China Antique'. (A) Morphological changes in lotus plumule during development. Bar = 1cm. (B) Length and (C) weight of lotus plumule at different developmental stages. Bars represent means ± standard error (*n* =3). (D) The alkaloid content in lotus plumule at different stages. Bars represent means ± standard error (*n* =4). Statistical significance is based on Least Significant Difference (LSD) test at $P < 0.05$, one-way ANOVA.

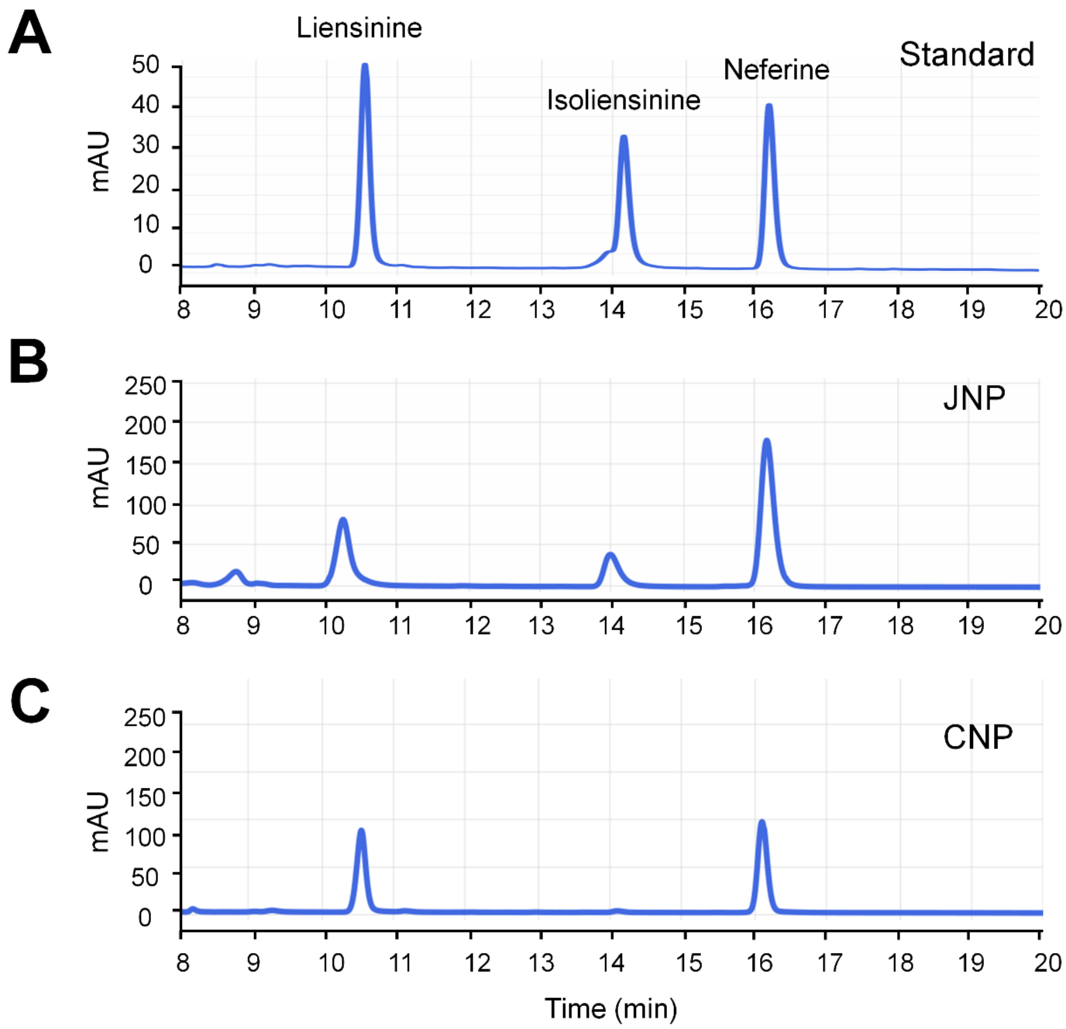

Supplementary Figure 2 HPLC scans of alkaloid components in lotus plumule. (A) HPLC scans of bisbenzylisoquinoline alkaloids standards. (B) and (C) Scans of alkaloid components in lotus plumule of cv. 'Jianxuan 17' (JNP) and 'China Antique' (CNP), respectively.

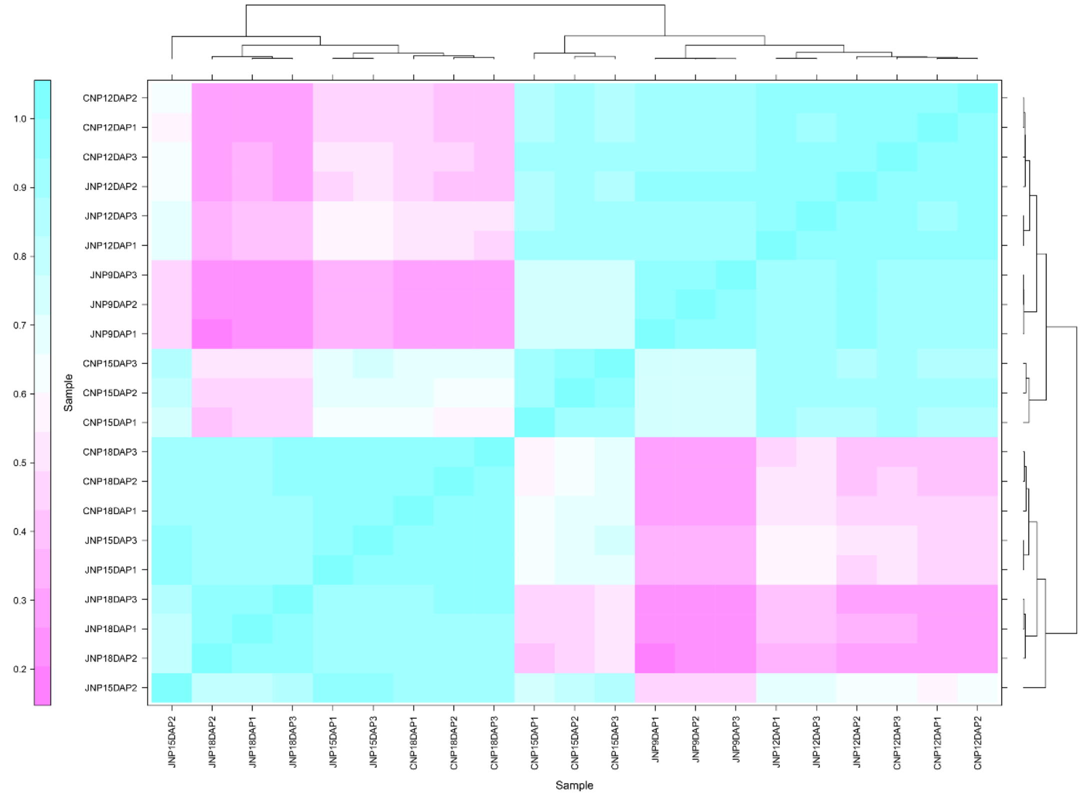

Supplementary Figure 3 Heat-map showing correlation coefficient matrix of samples.

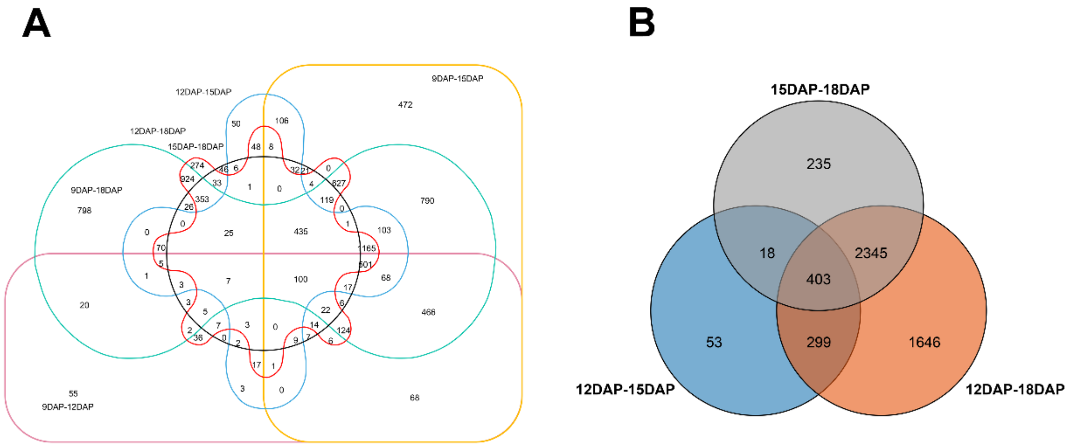

Supplementary Figure 4 Venn diagram showing overlap of DEGs in JNP (A) and CNP (B).

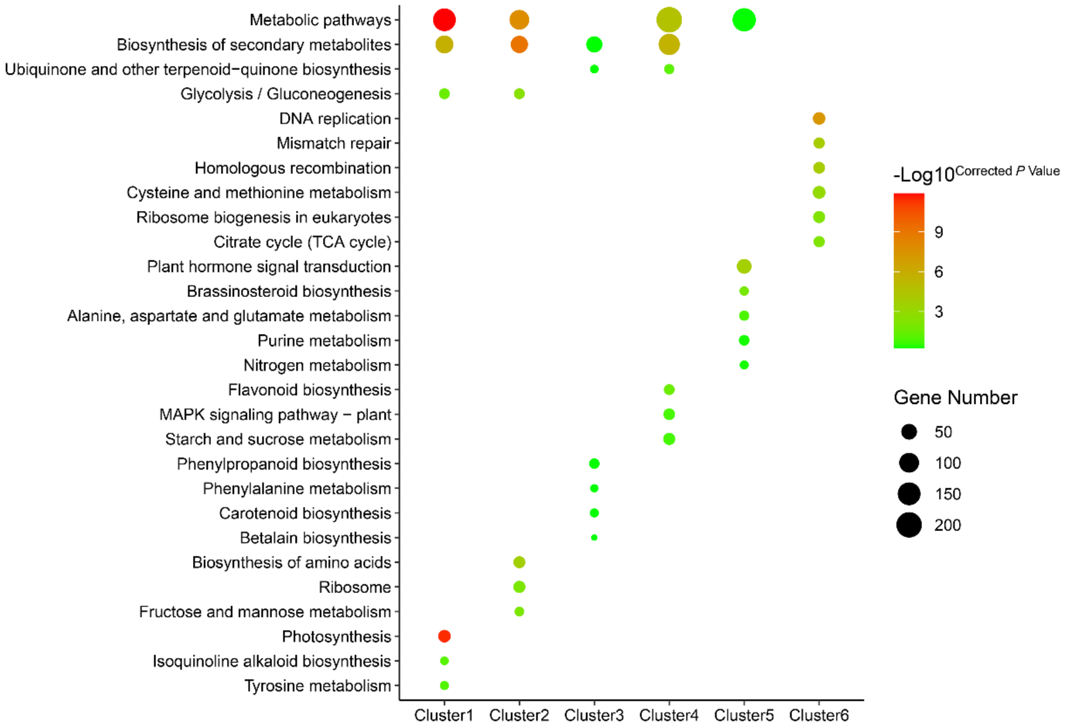

Supplementary Figure 5 KEGG analysis of DEGs in each subcluster.

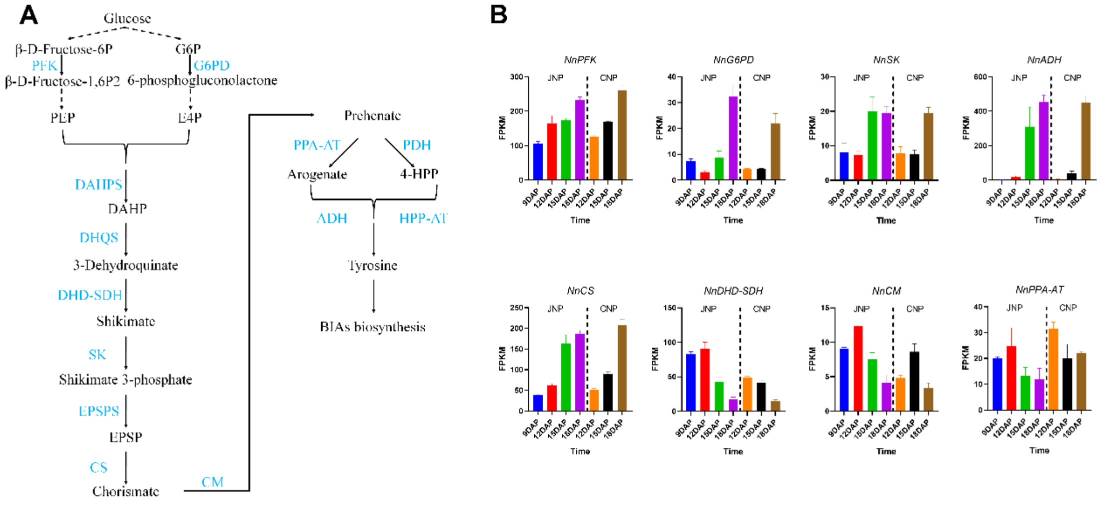

Supplementary Figure 6 Expression analysis of genes involved in tyrosine biosynthetic pathway. (A) Representative tyrosine biosynthetic pathway in plants. PFK, ATP-dependent 6-phosphofructokinase; G6PD, glucose 6-phosphate dehydrogenase; DAHPS, 3-deoxy-D-arabinoheptulosonate 7-phosphate synthase; DHQS, 3-dehydroquinate synthase; DHD, 3-dehydroquinate dehydratase; SDH, shikimate dehydrogenase; SK, shikimate kinase; EPSPS, 5-enolpyruvylshikimate-3-phosphate synthase; CS, chorismate synthase; CM, chorismate mutase; PPA-AT, prephenate aminotransferase; PDH, prephenate dehydrogenase; ADH, arogenate dehygrogenase; HPP-AT, 4-hydroxyphenylpyruvate aminotransferase. (B) Expression patterns of eight selected tyrosine biosynthesis genes. Bars represent means ± standard error (n =3). *NnPFK* (NNU_10589), *NnG6PD* (NNU_02159), *NnSK* (NNU_20134), *NnADH* (NNU_08507), *NnCS* (NNU_13158), *NnDHD-SDH* (NNU_06891), *NnPPA-AT* (NNU_20211), *NnCM* (NNU_04572).

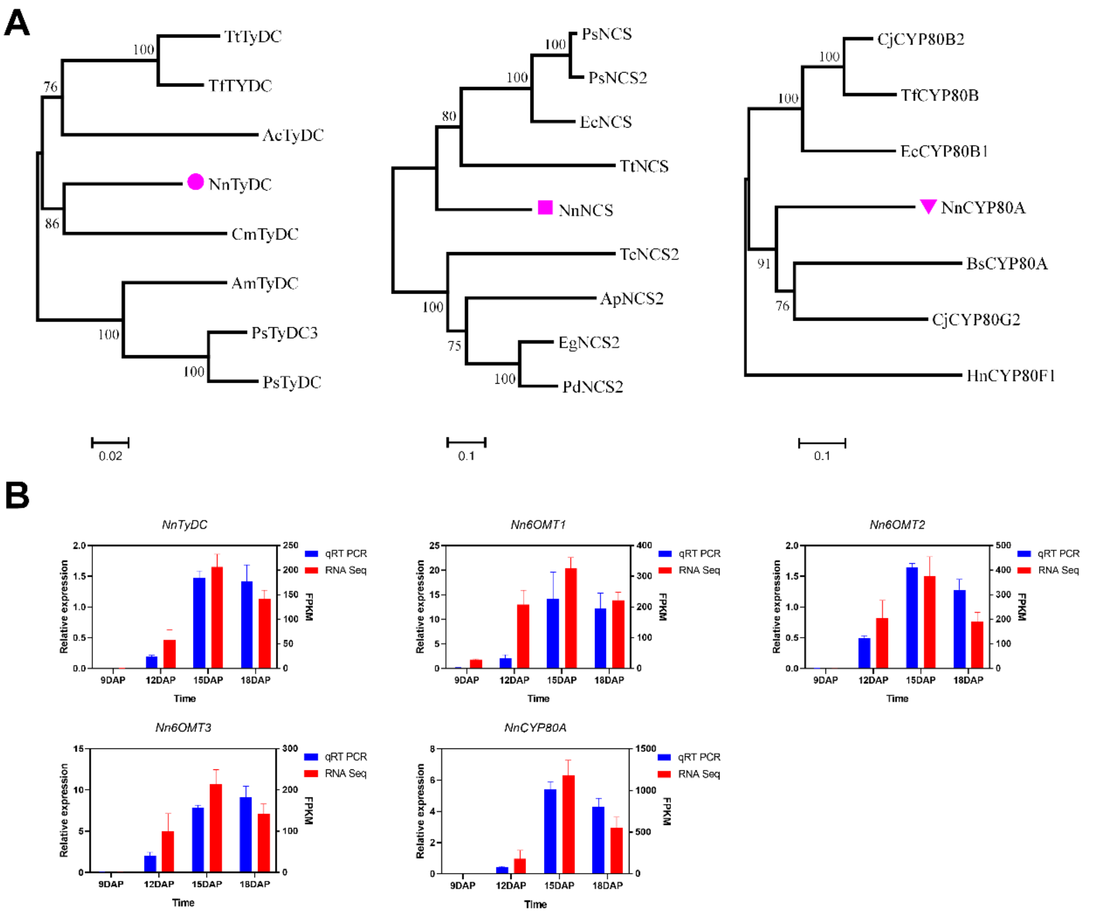

Supplementary Figure 7 Phylogenetic analysis and expression validation of bis-BIAs biosynthesis genes. (A) Phylogenetic relationship of NnTyDC (NNU_22559), NnNCS (NNU_14334), and NnCYP80A (NNU_21373). (B) qRT-PCR validation of RNA-Seq data using five bis-BIAs biosynthesis genes. *Nn6OMT1* (NNU_19035), *Nn6OMT2* (NNU_23168), *Nn6OMT3* (NNU_03166). Bars represent means ± standard error (n =3).

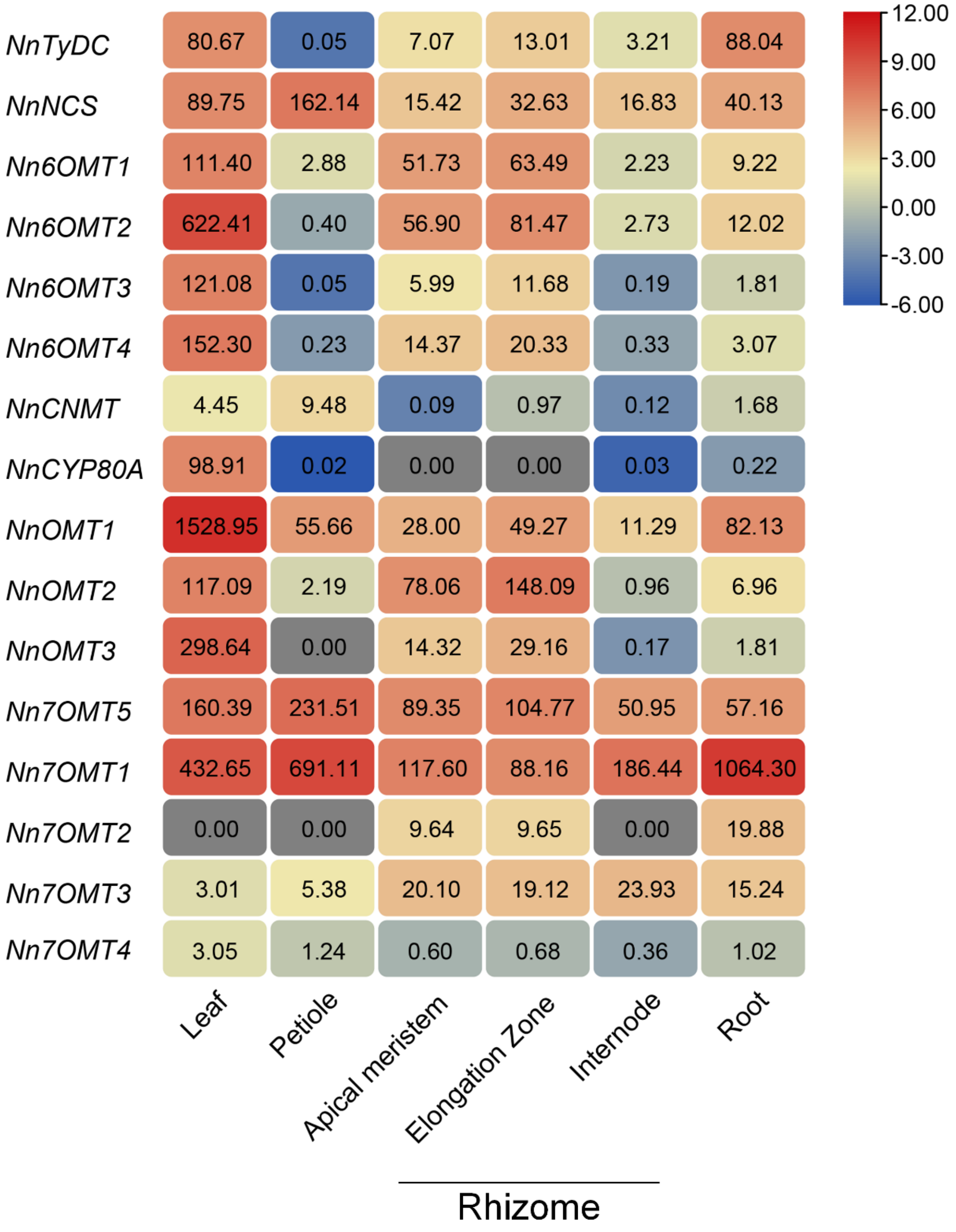

Supplementary Figure 8 Expression patterns of bis-BIAs biosynthesis genes in different tissues of cv. 'China Antique'.

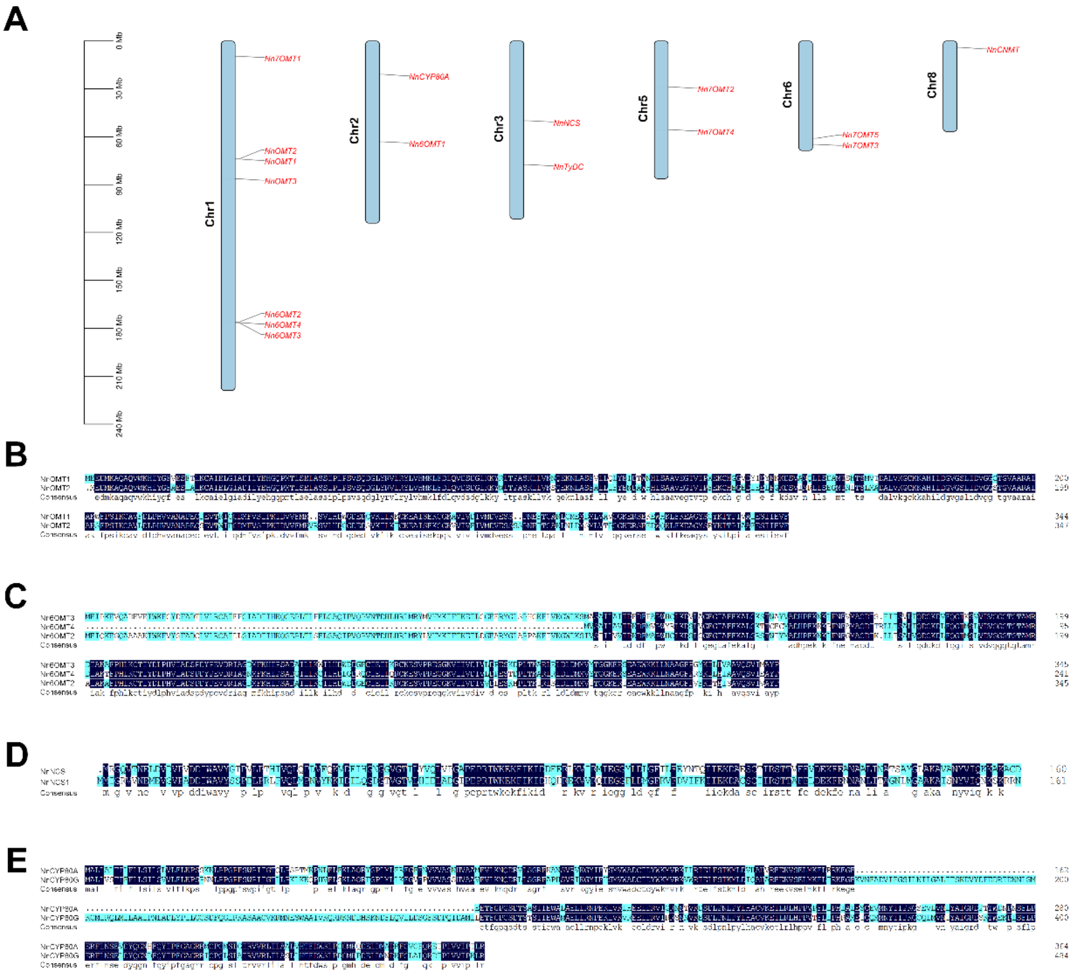

Supplementary Figure 9 Chromosomal localization and sequence alignment analysis of bis-BIAs biosynthesis genes. (A) Chromosomal localization of bis-BIAs biosynthesis genes in lotus. Alignments of NnOMT (B), Nn6OMT (C), NnNCS (D), and NnCYP80 (E) genes.

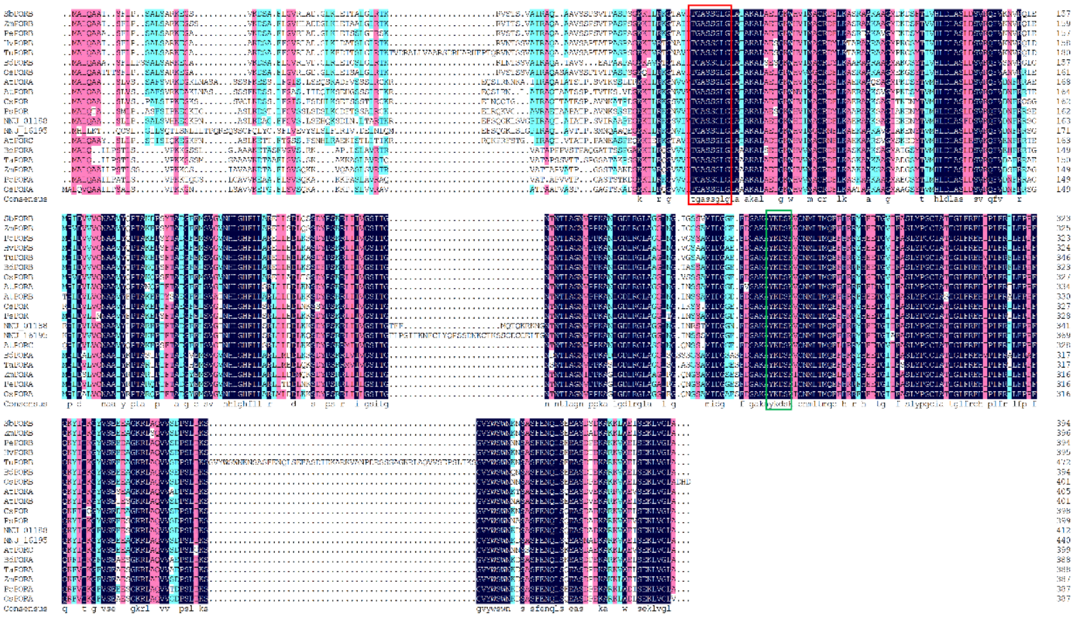

Supplementary Figure 10 Multiple alignment of POR amino acid sequences. The NADPH-binding motif (TGASSGLG) and the active site motif (YKDSK) are highlighted in red and green boxes, respectively.

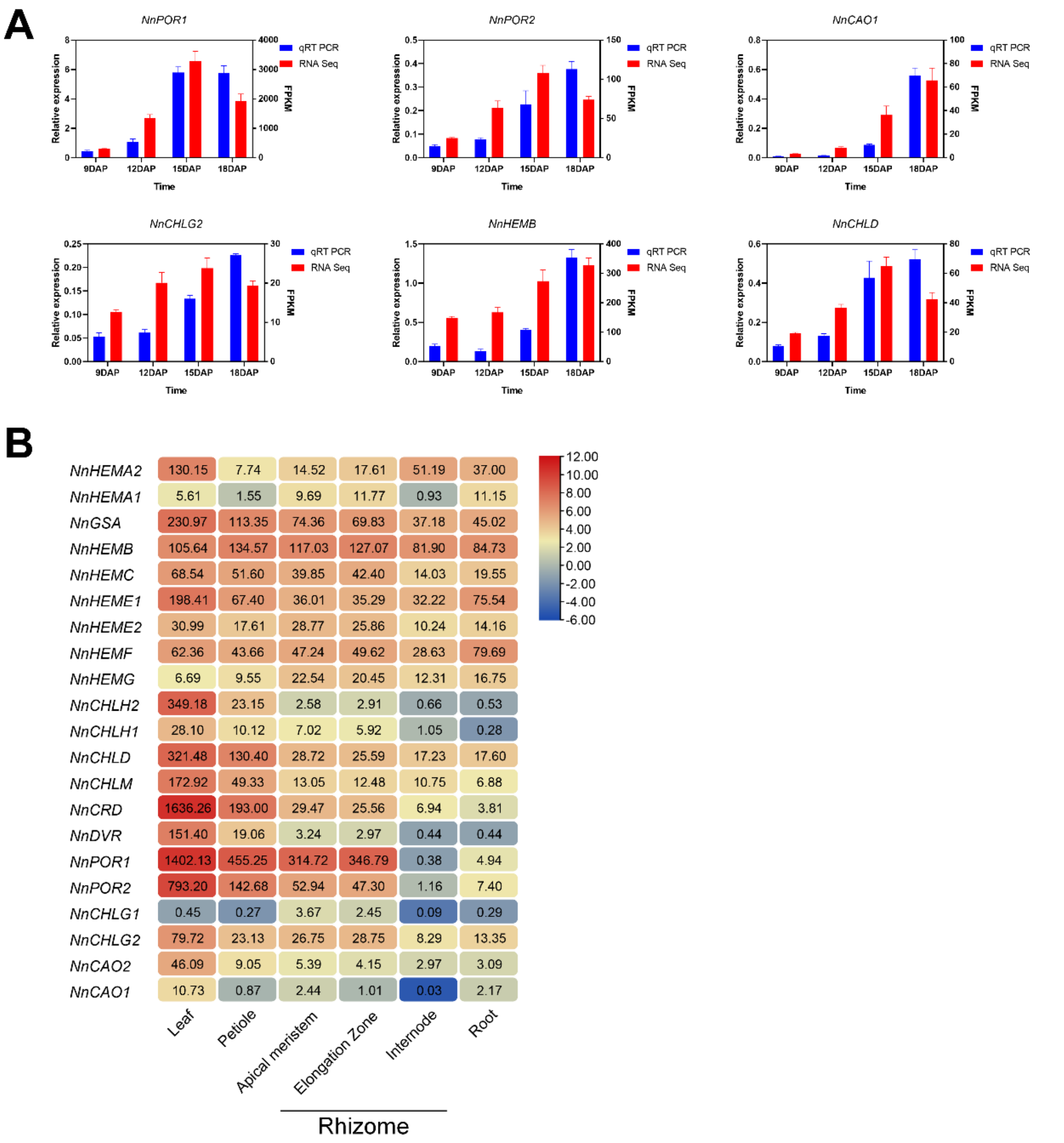

Supplementary Figure 11 Expression of Chl biosynthesis genes in lotus. (A) qRT-PCR validation of RNA-Seq data using six Chl biosynthesis genes. Bars represent means ± standard error (n =3). (B) Expression patterns of Chl biosynthesis genes in different tissues of cv. ‘China Antique’.

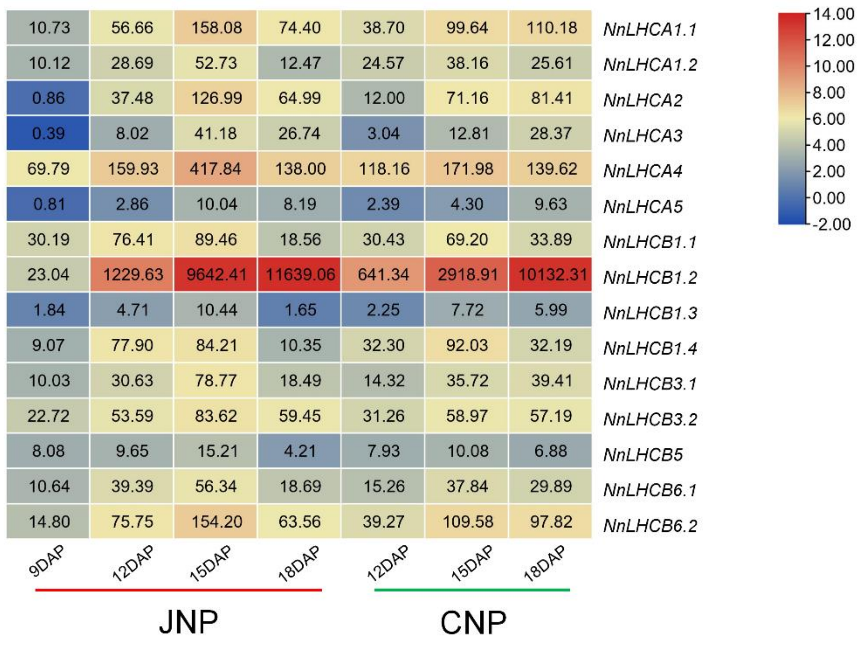

Supplementary Figure 12 Expression analysis of Light-harvesting chlorophyll-protein complex related genes during lotus plumule development.

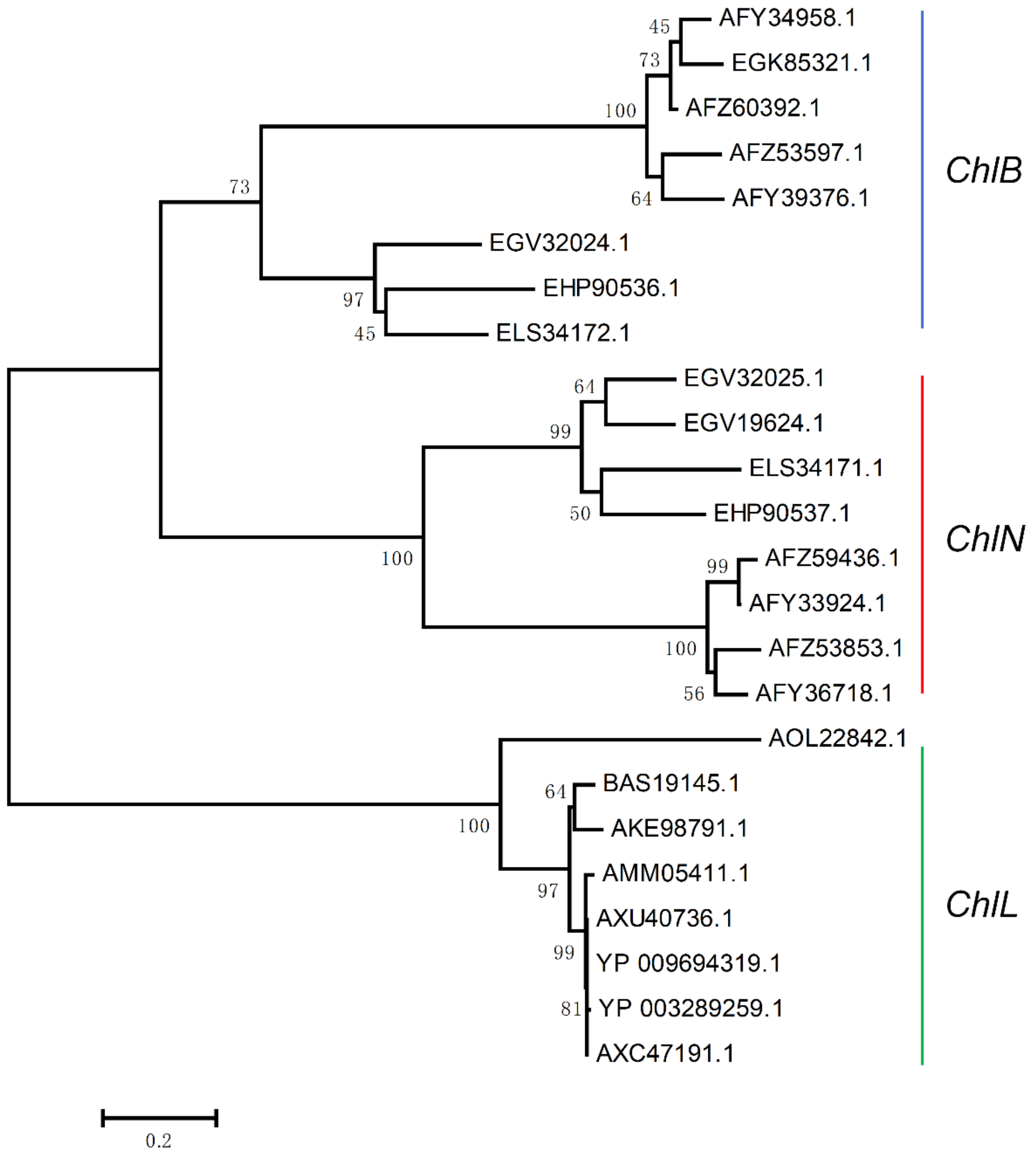

Supplementary Figure 13 Phylogenetic relationship of DROR genes used to identify DPOR genes in lotus by multiple sequences alignment. We used GenBank Accession Number as the name of DPOR genes.

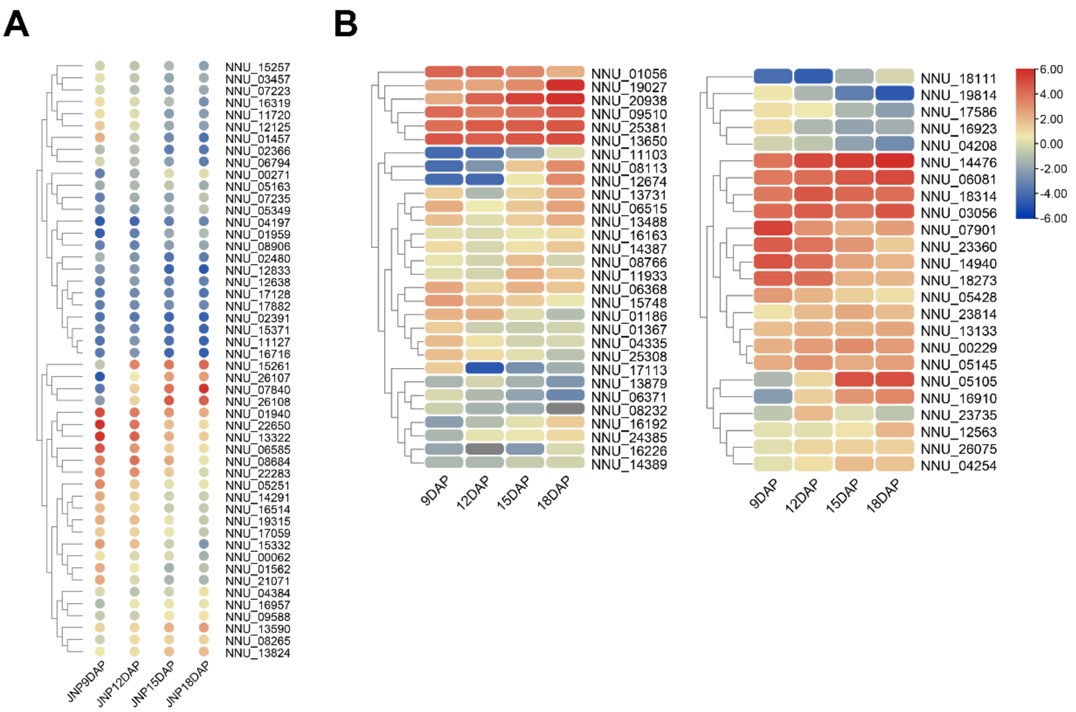

Supplementary Figure 14 Expression analysis of bHLH TFs and genes involved in the most enriched terms. (A) Expression patterns of differentially expressed bHLH genes. (B) Expression patterns of genes involved in ‘response to chitin’ (left) and ‘cell differentiation’ (right).

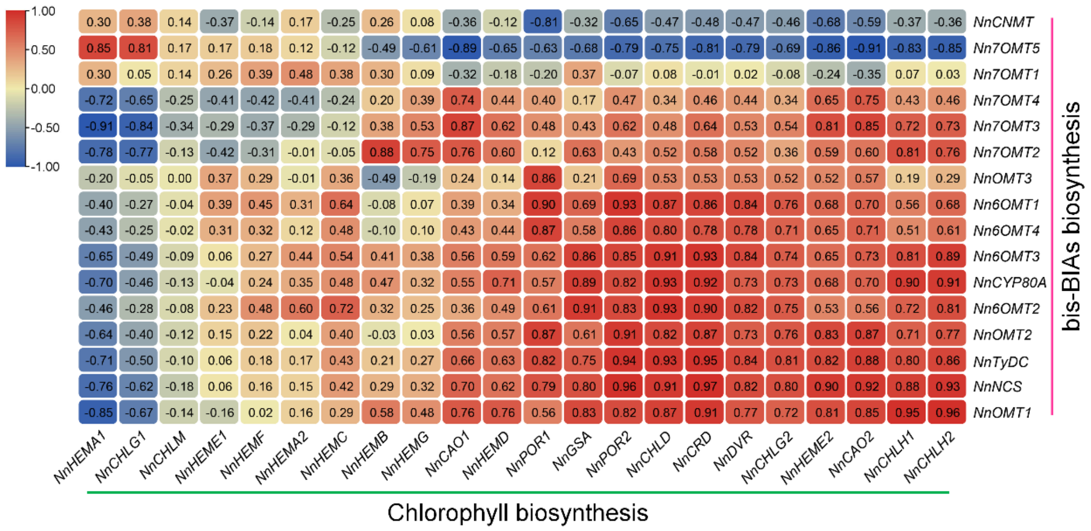

Supplementary Figure 15 Expression correlation analysis of genes related to bis-BIAs and chlorophyll biosynthesis in lotus plumule.
